# Genetic population structure and demography of an apex predator, the tiger shark *Galeocerdo cuvier*

**DOI:** 10.1002/ece3.5111

**Published:** 2019-05-04

**Authors:** Agathe Pirog, Sébastien Jaquemet, Virginie Ravigné, Geremy Cliff, Eric Clua, Bonnie J. Holmes, Nigel E. Hussey, John E. G. Nevill, Andrew J. Temple, Per Berggren, Laurent Vigliola, Hélène Magalon

**Affiliations:** ^1^ UMR ENTROPIE (Université de La Réunion/IRD/CNRS) Université de La Réunion Saint Denis, La Réunion France; ^2^ UMR PVBMT CIRAD St Pierre, La Réunion France; ^3^ KwaZulu‐Natal Sharks Board Umhlanga Rocks South Africa; ^4^ School of Life Sciences University of KwaZulu‐Natal Durban South Africa; ^5^ EPHE‐CNRS‐UPVD CNRS UPVD USR 3278 CRIOBE PSL Research University Perpignan France; ^6^ Laboratoire d'Excellence CORAIL Perpignan France; ^7^ School of Biological Sciences University of Queensland, St Lucia Brisbane Queensland Australia; ^8^ Biological Sciences University of Windsor Windsor Ontario Canada; ^9^ Environment Seychelles Victoria Seychelles; ^10^ School of Natural and Environmental Sciences Newcastle University Newcastle‐upon‐Tyne UK; ^11^ UMR ENTROPIE (Université de La Réunion/IRD/CNRS) Institut de Recherche pour le Développement Nouméa Nouvelle Calédonie France

**Keywords:** approximate Bayesian computation, bottleneck, effective population size, microsatellite DNA, mitochondrial DNA, tiger shark

## Abstract

Population genetics has been increasingly applied to study large sharks over the last decade. Whilst large shark species are often difficult to study with direct methods, improved knowledge is needed for both population management and conservation, especially for species vulnerable to anthropogenic and climatic impacts. The tiger shark, *Galeocerdo cuvier*, is an apex predator known to play important direct and indirect roles in tropical and subtropical marine ecosystems. While the global and Indo‐West Pacific population genetic structure of this species has recently been investigated, questions remain over population structure and demographic history within the western Indian (WIO) and within the western Pacific Oceans (WPO). To address the knowledge gap in tiger shark regional population structures, the genetic diversity of 286 individuals sampled in seven localities was investigated using 27 microsatellite loci and three mitochondrial genes (*CR*,*COI,* and *cytb*). A weak genetic differentiation was observed between the WIO and the WPO, suggesting high genetic connectivity. This result agrees with previous studies and highlights the importance of the pelagic behavior of this species to ensure gene flow. Using approximate Bayesian computation to couple information from both nuclear and mitochondrial markers, evidence of a recent bottleneck in the Holocene (2,000–3,000 years ago) was found, which is the most probable cause for the low genetic diversity observed. A contemporary effective population size as low as 111 [43,369] was estimated during the bottleneck. Together, these results indicate low genetic diversity that may reflect a vulnerable population sensitive to regional pressures. Conservation measures are thus needed to protect a species that is classified as Near Threatened.

## 1 INTRODUCTION

Study of large sharks, including the tiger shark *Galeocerdo cuvier,* the great white shark *Carcharodon carcharias,* and the whale shark *Rhincodon typus*, is challenging as these species spend substantial periods of their lifetime in open ocean waters. Consequently, data concerning basic aspects of their biology, such as migration patterns and population structure, are limited (Conrath, Musick, Carrier, & Heithaus, 2012; Musick, 2010). Understanding the ecological role of large sharks in marine ecosystems also remains incomplete. However, as apex predators they are considered to exercise important functions in marine food webs via top‐down processes (Dudley & Simpfendorfer, 2006; Ferretti, Worm, Britten, Heithaus, & Lotze, 2010; Myers, Baum, Shepherd, Powers, & Peterson, 2007). Large sharks typically present classically *K*‐selected life histories, with slow growth rate, late maturity, and low fecundity (Musick, Burgess, Cailliet, Camhi, & Fordham, 2000). This renders them vulnerable to overexploitation with low rebound potentials limiting their recovery (Campana & Ferretti, 2016; Cortés, 2002; Dudley & Simpfendorfer, 2006; Ferretti et al., 2010; Myers & Worm, 2005; Worm et al., 2013). Certain species, including the white, tiger, and bull sharks, are also responsible for the majority of human–shark conflicts, complicating conservation and management actions. Consequently, continuing to build on our current understanding of the biology and the ecology of these large sharks is needed to facilitate appropriate management .

The tiger shark is a large (up to 5.5 m long) Carcharhinid (Meyer et al., 2014) with a circumglobal distribution in tropical and subtropical waters (Compagno, 1984, 1990). It is a potential keystone species in marine ecosystems through predation or by inducing behavioral modifications of its prey, and thus indirectly modifying primary producer community structure, biomass, and nutrient composition (Burkholder, Heithaus, Fourqurean, Wirsing, & Dill, 2013; Heithaus, Frid, Wirsing, & Worm, 2008; Wirsing, Heithaus, & Dill, 2007). The tiger shark is listed as globally “Near Threatened” by the International Union for Conservation of Nature (IUCN) Red List of Threatened Species and is primarily threatened by fisheries exploitation (Simpfendorfer, 2009; Temple et al., 2018). Clarke et al. (2006) estimated that ~400,000–500,000 tiger sharks are caught annually for the shark fin trade globally. Furthermore, this species is currently targeted by shark control programmes in the Indo‐Pacific: South Africa (Cliff & Dudley, 1991; Dudley, 1997; Sumpton, Taylor, Gribble, McPherson, & Ham, 2011) and Australia (Holmes et al., 2012; Reid & Krogh, 1992; Simpfendorfer, 1992), and formerly in Hawaii (Wetherbee, Lowe, & Crow, 1994). It is also reported as bycatch in pelagic fisheries, in the Western Pacific (Polovina & Lau, 1993) and in the southern (Afonso & Hazin, 2014) and northwestern Atlantic (Baum et al., 2003). Trends in long‐term catch and catch rates are difficult to obtain for sharks not specifically targeted by fisheries. Nevertheless, long‐standing control programmes as well as logbooks from the longline fishing fleets do provide long‐term data using standardized fishing methods, which may be used to assess catch per unit effort trends over time. Tiger shark catch rates in control programmes appear to be increasing in KwaZulu‐Natal, South Africa (Dudley & Simpfendorfer, 2006), while contrasting declines were observed in Queensland (Holmes et al., 2012), New South Wales, Australia (Reid, Robbins, & Peddemors, 2011), and in commercial fisheries in the northern Atlantic (Baum et al., 2003; Myers et al., 2007). These differences in catch rates indicate regional variation in population trends of tiger sharks, but overall evidence supports declining populations. Control programme catch rates coupled with mark‐recapture and telemetry also provide information on habitat use patterns.

Data from pelagic longline fisheries have highlighted the importance of the pelagic realm to a species originally described as coastal (Domingo et al., 2016; Polovina & Lau, 1993). More recently, tiger shark individuals have been recorded moving over several thousands of kilometers distances (Ferreira et al., 2015; Hammerschlag, Gallagher, Wester, Luo, & Ault, 2012; Holmes et al., 2014; Lea et al., 2015; Werry et al., 2014), including crossing ocean basins (Afonso, Garla, & Hazin, 2017; Heithaus, Wirsing, Dill, & Heithaus, 2007; Kohler, Casey, & Turner, 1998; Kohler & Turner, 2001). Equally, tracking studies have also revealed strong residency patterns, with some individuals maintaining large but defined home ranges and returning to specific locations on a regular basis (Ferreira et al., 2015; Fitzpatrick et al., 2012; Heithaus, 2001; Holland, Wetherbee, Lowe, & Meyer, 1999; Lowe, Wetherbee, & Meyer, 2006). These patterns seem to be linked not only to intrinsic states such as foraging strategies (Heithaus, Hamilton, Wirsing, & Dill, 2006; Holland et al., 1999; Meyer, Clark, Papastamatiou, Whitney, & Holland, 2009; Meyer, Papastamatiou, & Holland, 2010; Papastamatiou et al., 2011) and sex (Heithaus et al., 2006; Papastamatiou et al., 2013; Sulikowski et al., 2016) but also to extrinsic drivers, notably prey abundance (Lowe, Wetherbee, Crow, & Tester, 1996) and water temperature (Heithaus, 2001; Holmes et al., 2014; Wirsing, Heithaus, & Dill, 2006). While these movement studies highlight the complex migration patterns and habitat use of tiger sharks, the implications of these patterns on population connectivity and structure have only begun to be considered recently using molecular markers.

The first study investigating the population genetic structure of the tiger shark was primarily designed for species delimitation examining 29 samples collected throughout its range using the mitochondrial NADH dehydrogenase subunit (*ND2*) gene (Naylor et al., 2012). Two monophyletic clades were identified, one in the Atlantic and the other in the Indo‐Pacific, with no shared haplotype, suggesting the presence of two subspecies. This hypothesis was subsequently refuted by Bernard et al. (2016), using a larger sample of 380 individuals from several sampling sites across the three ocean basins, 10 microsatellite loci and two mitochondrial genes, the control region (*CR*) and the cytochrome oxidase c subunit I (*COI*). Bernard et al. (2016) highlighted long‐term genetic isolation between tiger shark populations of the Atlantic and the Indo‐Pacific, but with shared mitochondrial haplotypes, which is inconsistent with the two subspecies hypothesis. Furthermore, samples from Hawaii appeared genetically differentiated from all other locations, perhaps due to more restrictive movement behaviors and greater residency exhibited by sharks from this area (Meyer et al., 2010; Papastamatiou et al., 2013). Using mitochondrial data, Bernard et al. (2016) also identified a larger degree of genetic differentiation in the Indo‐Pacific than observed with microsatellite data. Recorded differences between the west and east coast of Australia were hypothesized to be a result of a stronger matrilineal structure due to sex‐biased dispersal and female philopatry. An additional study focused on the population structure of tiger sharks across the eastern Indian Ocean and the Pacific, with 355 samples collected primarily around Australia and Hawaii, with eight samples from Brazil in the southwest Atlantic as an outgroup (Holmes et al., 2017). Using nine microsatellite loci, Holmes et al. (2017) confirmed genetic isolation between the western Atlantic and the Indo‐Pacific. Further, the high connectivity around Australia found by Bernard et al. (2016) was reflected in satellite tracking data (Afonso et al., 2017; Heithaus et al., 2007; Kohler et al., 1998). Nevertheless, contrary to the findings of Bernard et al. (2016), Holmes et al. (2017) found no genetic differentiation between Hawaiian and Australian populations.

In the present study, we used samples from the western Indian Ocean (233 individuals from four locations), the eastern Indian Ocean (nine individuals from one location), and the western Pacific (33 individuals from two locations), and 27 microsatellite loci and three mitochondrial genes (*CR*,* COI,* and *cytb*) to further investigate population structure and demographic parameters in the tiger shark. As we used samples in common with Holmes et al. (2017) and the nine microsatellite loci they used, we were able to combine samples to obtain a more precise picture of the population genetic dynamics of the tiger shark in the Indo‐Pacific. In addition, *CR* sequences obtained by Bernard et al. (2016) were used in conjunction with our data, to further investigate their proposed mitochondrial structure in these oceans.

## 2 MATERIALS AND METHODS

### 2.1 Sampling

**Figure 1:**
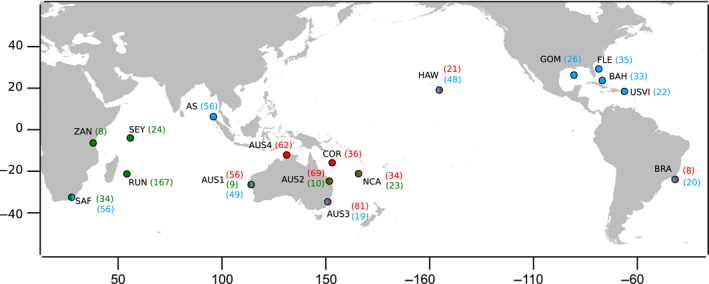
Map of tiger shark (*Galeocerdo cuvier*) sampling locations (AS: Andaman Sea; AUS1: Western Australian coast; AUS2: Queensland, Australia; AUS3: New South Wales, Australia; AUS4: Northern Territories, Australia; BAH: Bahamas; COR; BRA: Brazil; Coral Sea; FLE: Florida East Coast; HAW: Hawaii; GOM: Gulf of Mexico; NCA: New Caledonia; RUN: Reunion Island; SEY: Seychelles; SAF: South Africa;USVI:US Virgin Islands; ZAN: Zanzibar). In brackets are sample sizes. In green are indicated samples collected for this study and genotyped with 27 microsatellite loci and three mitochondrial genes. Red indicates samples genotyped by Holmes et al. (2017) with the nine microsatellite loci developed by Bernard et al. (2015). Blue indicates samples sequenced at the control region by Bernard et al. (2016)

Samples were collected in four locations in the western Indian Ocean (Zanzibar, ZAN: *n = *8; South Africa, SAF: *n = *34; the Seychelles, SEY: *n = *24; Reunion Island, RUN: *n = *167), and in the eastern Indian Ocean, along the Australian west coast (AUS1: *n = *9). Samples were collected in the western Pacific from the northeast coast of Australia (Queensland, AUS2: *n = *10) and in New Caledonia (NCA: *n *= 23; Figure 1). Samples came from individuals caught by fishermen or shark control programmes (fin clips or muscle tissue) and from scientific projects (i.e., biopsies) and were preserved in 90% ethanol.

### 2.2 Laboratory procedures

Genomic DNA was extracted using Qiagen DNeasy Blood & Tissue kit (Qiagen, Hilden, Germany) following manufacturer instructions.

The genotyping of all samples was performed at 30 microsatellite loci. Twenty‐six of them were species‐specific loci: the eight Gc‐loci developed by Pirog, Jaquemet, Blaison, Soria, and Magalon (2016), the nine TGR‐loci developed by Bernard, Feldheim, and Shivji (2015), and the nine TIG‐loci developed by Mendes et al. (2016). The remaining microsatellite loci were originally developed for the bull shark *Carcharhinus leucas* (Cl12, Cl14, and Cl17; Pirog, Blaison, Jaquemet, Soria, & Magalon, 2015) and the blacktip shark *Carcharhinus limbatus* (Cli100; Keeney & Heist, 2003), and successfully cross‐amplified in the tiger shark. The Gc‐loci as well as Cl12, Cl14, and Cl17 were directly fluorochrome‐labeled (using 6‐FAM, PET, VIC, or NED) and PCR reactions were performed following Pirog et al. (2016). Other loci were indirectly fluorochrome‐labeled (using 6‐FAM, PET, VIC or NED) and PCR reactions were performed following Gélin, Postaire, Fauvelot, and Magalon (2017). All loci were multiplexed post‐PCR in five panels (Appendix S1). PCR products were genotyped using an ABI 3730XL capillary sequencer at the Plateforme Gentyane (INRA, Clermont‐Ferrand, France). Allelic sizes were determined with genemapper v.4.0 (Applied Biosystems) using an internal size standard (Genescan LIZ‐500, Applied Biosystems). Of the nine loci developed by Bernard et al. (2015), we did not keep TGR233 as we found it difficult to read. Of the loci developed by Mendes et al. (2016), we did not keep either TIG05, as it was difficult to read with several failed amplifications, or TIG25 as it was monomorphic throughout our samples. We thus kept 27 microsatellite loci for further analyses.

Microsatellite genotypes generated by Holmes et al. (2017) and Bernard et al. (2016) were also added to the present study (Figure 1; Table 1). Individuals from Australia genotyped in this study are the same as those in Holmes et al. (2017), which were genotyped with the nine microsatellite loci developed by Bernard et al. (2015). For these individuals and the eight microsatellites in common between the present study and Holmes et al. (2017), the genotypes were compared and allele lengths calibrated. To ensure the allele frequency bins were uniform between the studies at each locus, alleles frequencies were plotted and compared for each sampling location (Appendix S2). We thus added genotypes of all the individuals from Holmes et al. (2017) (Figure 1, Table 1) enlarging the geographic coverage of our sampling: northern and southeastern Australian coasts, Coral Sea, Hawaii, and Brazil. We also expanded the number of individuals for some locations in common: western (AUS1) and northeastern (AUS2) Australian coasts, and New Caledonia (Figure 1, Table 1).

**Table 1:** Summary of *Galeocerdo cuvier* sampling locations and number of individuals from this study, Holmes et al. (2017) and Bernard et al. (2016), as well as the molecular markers used in each study and the different datasets analyzed in the present study

	This study	Holmes et al. (2017)	Bernard et al. (2016)
Sampling locations			
Western Indian Ocean			
ZAN	8		
SEY	24		55
SAF	34		
RUN	167		
Eastern Indian Ocean			
AS			31
AUS1	**9**	**9 **+** **47	66
Western Pacific Ocean			
AUS4		62	
COR		36	
AUS2	**10**	**10 **+** **59	
AUS3		81	21
NCA	23	22	
Central Pacific			
HAW		21	65
Northwestern Atlantic			
GOM			26
FLE			35
BAH			39
USVI			22
Southwestern Atlantic			
BRA		8	20
Microsatellite loci	Cl12; Cl14; Cl17^a^ Gc01‐Gc08^b^		
	**Cli100** a		**Cli100**
	TIG01; TIG07; TIG10; TIG12; TIG15; TIG17; TIG19^d^		
	**TGR47; TGR212; TGR348; TGR891; TGR943; TGR1033; TGR1157; TGR1185** e		
		TGR233	
Mitochondrial loci	***CR; COI***		***CR; COI***
	*cytb*		
Microsatellite datasets	27‐msat (*n* = 275)	Holmes 8‐msat (*n *= 355)	Bernard 8‐msat (*n* = 380)
	8‐msat (*n* = 606)		
Mitochondrial datasets	*CR‐COI‐cytb* (*n* = 127)		
	*CR* (*n *= 538)		*CR* (*n* = 538)

Notes

In bold are indicated samples or markers in common in several studies.

Pirog et al. (2015).

Pirog et al. (2016).

a Keeney and Heist (2003).

Mendes et al. (2016).

Bernard et al. (2015).

Moreover, for DAPC analyses (see below) we also used the microsatellite genotypes generated by Bernard et al. (2016), retaining only the eight microsatellites in common with our study and Holmes et al. (2017). However, we could not analyze them together with our genotypes as we did not have in our possession samples from individuals in common to calibrate the electrophoregrams.

In summary, four microsatellite datasets were used (Table 1): (a) 27‐msat dataset (the 27 microsatellites on the individuals from this study only; *n* = 275), (b) 8‐msat dataset (the 8 microsatellites in common for all studies on the individuals from this study and those from Holmes et al. (2017) only; *n* = 606), (c) Holmes 8‐msat dataset (the 8 microsatellites in common for all studies on the individuals from Holmes et al. (2017) only; *n* = 355), and (4) Bernard 8‐msat dataset (the 8 microsatellites in common for all studies on the individuals from Bernard et al. (2016) only; *n* = 380).

The mitochondrial DNA control region (*CR*) was amplified using the set of primers Gc‐CR‐F/Gc‐CR‐R (Pirog et al., 2016), the cytochrome oxidase c subunit I (*COI*) using the primer cocktails C_FishF1t1/C_FishR1t1 (Ivanova, Zemlak, Hanner, & Hebert, 2007; Ward & Myers, 2005) as described in Wong, Shivji, and Hanner (2009) and the cytochrome b (*cytb*) using GluDG/C61121H (Naylor, Ryburn, Fedrigo, & Lopez, 2005). This was performed for subsets of the whole dataset: 200 individuals for *CR*, 147 individuals for *COI,* and 130 for *cytb*. Primers were used for both amplification and direct sequencing. PCR reactions were performed in a total volume of 25 μl: 1× of MasterMix (Applied Biosystems), 0.3 μM of forward and reverse primers/cocktails, and 1.6 ng/μL of genomic DNA. The thermocycling programme for *CR* is described in Pirog et al. (2016). For *COI* and *cytb*, the same programme was used, except that the PCR annealing temperature was set at 53°C. Amplicons were sent for sequencing to Genoscreen (Lille, France) on a capillary sequencer ABI 3730XL (Applied Biosystems).

Moreover, to complement the mitochondrial analyses, *CR* sequences generated by Bernard et al. (2016) (GenBank accession numbers: KU847364–KU847386) were added to our dataset (named hereafter the *CR* dataset; *n* = 538), adding samples from 10 locations for *CR* analyses (Figure 1; Table 1): Gulf of Mexico, Florida East Coast, Bahamas, US Virgin Islands, Brazil (referred to as western South Atlantic therein), South Africa (western South Indian Ocean therein), Andaman Sea, Western Australia (eastern South Indian Ocean therein), southeastern Australia (western South Pacific Ocean therein), and Hawaii (Central Pacific Ocean therein).

### 2.3 Genetic diversity analyses

Null alleles and allelic drop‐out occurrence and frequencies were assessed using microchecker v.2.2.3 (Van Oosterhout, Hutchinson, Wills, & Shipley, 2004). Linkage disequilibrium (LD) between pairs of loci was tested using a likelihood‐ratio test with 10,000 permutations in arlequin v.3.5.1.2 (Excoffier & Lischer, 2010). Diversity indices such as the number of alleles per locus *N*_a_, observed and expected heterozygosities (*H*_O_ and *H*_E_) and inbreeding coefficient *F*_IS_ (Weir & Cockerham, 1984) were assessed using fstat v.2.9.3.2 (Goudet, 1995). Departure from Hardy–Weinberg equilibrium (HWE) was tested using 5,000 permutations in fstat v.2.9.3.2 (Goudet, 1995). The mean allelic richness *A*_r_ and the mean private allelic richness *A*_rp_ were calculated using a rarefaction method, as implemented in HP‐rare v.1.0 (Kalinowski, 2005). This method accounts for differences in sample size by standardizing *A*_r_ and *A*_rp_ values across sampled locations by resampling the lowest number of genotypes available (i.e., 16 haploid gene copies or eight diploid genotypes in Zanzibar) in each location.

Mitochondrial sequences were quality checked and aligned using geneious v.8.1.2 (Kearse et al., 2012). Alignments were performed using the MAFFT method (Katoh, Misawa, Kuma, & Miyata, 2002), first for each gene separately and then for the concatenated sequence *CR‐COI‐cytb*. Molecular diversity indices (i.e., number of haplotypes, number of segregating sites, haplotype (*h*) and nucleotide (π) diversities) were calculated for each region separately and for the concatenated dataset (called hereafter *CR‐COI‐cytb* dataset), using dnaSP v.5.10.1 (Librado & Rozas, 2009).

Detection of partitioning schemes and substitution models within the concatenated sequence *CR‐COI‐cytb* was performed using partitionfinder v.2.1.1 (Guindon et al., 2010; Lanfear, Frandsen, Wright, Senfeld, & Calcott, 2017). beast v.1.8.4 (Drummond, Suchard, Xie, & Rambaut, 2012) was used to reconstruct phylogenetic relationships on the *CR* dataset, and on the *CR‐COI‐cytb* dataset. Bayesian Markov chain Monte Carlo (MCMC) analyses were performed assuming a HKY85 model of substitution as the latter was shown to best fit the data (see Section 3). Rate of variation among sites was modeled with a discrete gamma distribution with four rate categories. We assumed an uncorrelated lognormal relaxed clock to account for rate variation among lineages. To minimize prior assumptions about demographic history, we adopted an extended Bayesian skyline plot (EBSP) approach in order to integrate data over different coalescent histories. Evolutionary model parameters were then estimated, with samples drawn from the posterior every 10^5^ MCMC steps over a total of 10^8^ steps from five independent runs. The first 10^7^ steps were discarded as burn‐in. Good mixing and convergence were assessed using tracer v.1.6 (Rambaut, Suchard, Xie, & Drummond, 2014) and the best tree was selected using the maximum clade credibility option with treeannotator v.1.8.4 (Drummond et al., 2012) and viewed with figtree v.1.4.0 (http://tree.bio.ed.ac.uk/software/figtree/). To further evaluate phylogenetic relations among haplotypes, TCS statistical parsimony networks (Clement, Posada, & Crandall, 2000) were constructed using popart v.1.7 (Leigh & Bryant, 2015).

### 2.4 Population genetic structure

Two complementary clustering methods were used to investigate population structure in the tiger shark. First, Bayesian clustering analyses were performed using Structure v.2.3.4 (Falush, Stephens, & Pritchard, 2003; Pritchard, Stephens, & Donnelly, 2000). For any given number of clusters (*K*) between 1 and 10, individual assignment probabilities to each cluster were determined so as to minimize departures from HWE within clusters and maximize LD among clusters. Two analyses were performed, with and without the LOCPRIOR model, which uses prior sampling location information in the Bayesian clustering, to allow detection of weaker genetic population structure (Hubisz, Falush, Stephens, & Pritchard, 2009). Conditions were set to 10^6^ chain length after a burn‐in of 5 × 10^5^ and 10 chains were run for each *K*, assuming correlated allele frequencies and the admixture model. The analysis was also performed using the alternative ancestry prior as suggested in Wang (2017), but it did not alter the results. For a given *K*, distinct modes were identified and, for each mode and each individual, the assignment probabilities to each cluster were averaged using clumpak (Kopelman, Mayzel, Jakobsson, Rosenberg, & Mayrose, 2015). These analyses were performed on both 27msat and 8msat datasets. Secondly, a Discriminant Analysis of Principal Components (DAPC; Jombart, Devillard, & Balloux, 2010), which in contrast to structure does not rely on HWE or LD to identify clusters, was performed to check consistency between clustering methods based on different algorithms. This method transforms genotypes using PCA as a prior step to a discriminant analysis and defines clusters by minimizing variations within while maximizing differentiation among clusters. DAPC was applied using the *adegenet* package (Jombart, 2008) for R (R Development Core Team 2017). We tested values of *K* ranging from 1 to 50 and visualized the Bayesian Information Criterion (BIC) values for increasing *K* using the find.clusters() function. We then used the dapc() function for values of *K* ranging from 1 to 10, retaining a number of principal components sufficient to explain ≥90% of total variance of the data (Jombart et al., 2010). This analysis was performed on the four microsatellite datasets (see Laboratory Procedures above). Methods traditionally used to detect the most likely number of clusters within a dataset (Pr(X/*K*), Pritchard et al. 2000; the Δ*K* method, Evanno, Regnaut, & Goudet, 2005; the Deviance Information Criterion (DIC), Gao, Bryc, & Bustamante, 2011; the Bayesian Information Criterion (BIC), Jombart et al., 2010; the Thermodynamics Integration (TI), Verity & Nichols, 2016) may provide different outputs and do not always reflect the biological truth. To cope with these inconsistencies, we tested different *K*‐values and different numbers of clusters in structure and DAPC, and chose to consider the number of clusters and the individual assignments that were retrieved by both types of analyses and that seemed biologically meaningful.

Assessing population differentiation between pairs of sampling locations, *F*_ST_ (Weir & Cockerham, 1984) and *D*_est_ (Jost, 2008) were estimated for the microsatellite data with arlequin v.3.5.1.2 (Excoffier & Lischer, 2010) and *DEMEtics* v.0.8‐7 (Gerlach, Jueterbock, Kraemer, Deppermann, & Harmand, 2010), respectively, on both 27msat and 8msat datasets. Contrary to *F*_ST_, which depends on within‐population diversity and is affected by migration rates and effective population sizes, *D*_est_, based on the effective number of alleles strictly reflects the differentiation between populations. For the *CR‐COI‐cytb* dataset as well as the *CR* dataset, the metric Φ_ST_ (Slatkin, 1995) was estimated using arlequin v.3.5.1.2 (Excoffier & Lischer, 2010). Significance of pairwise population differentiation indices was tested using 10,000 permutations.

### 2.5 Population demography

To test for departures from a constant population size (Ramos‐Onsins & Rozas, 2000), the summary statistics Tajima's *D* (Tajima, 1989) and Fu's *F*_S_ (Fu, 1997) were estimated from the *CR‐COI‐cytb* dataset with arlequin v.3.5.1.2 (Excoffier & Lischer, 2010), with significance tested implementing 10^5^ simulated samples.

**Figure 2:**
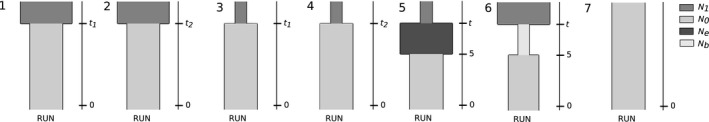
Graphical representations of the seven scenarios depicting possible variations in effective population size of *Galeocerdo cuvier* population, using individuals from Reunion Island (RUN). The time was measured backward in generations before present. *N*_0_, the actual effective population size; *N*_1_, the ancestral effective population size; *N*_b_, the effective population size during a bottleneck; *N*_e_, the effective population size during an expansion; *t*_1_, beginning of decrease or expansion for Scenarios 1 and 3; *t*_2_, beginning of decrease or expansion for Scenarios 2 and 4; *t*, beginning of the expansion or bottleneck period for Scenarios 5 and 6

Furthermore, to identify effective population size variations in the Indo‐Pacific, as no population structure was highlighted between both the western Indian and the western Pacific Oceans, a coalescent framework was used through approximate Bayesian computation (ABC) using diyabc v.2.1.0 (Cornuet et al., 2014) with both the 27‐msat dataset and the *CR‐COI‐cytb* dataset. We defined *N*_0_ the actual effective population size, *N*_1_ the ancestral effective population size, *N*_b_ the effective population size during a bottleneck, and *N*_e_ the effective population size during an expansion. Then, seven scenarios (Figure 2) were tested: (Scenario 1) a recent (<500 generations) decrease (*N*_0_ < *N*_1_), (Scenario 2) a more ancient (between 10^3^ and 5 × 10^5^ generations in the past) decrease, (Scenario 3) a recent (less than 500 generations) expansion (*N*_0_ > *N*_1_), (Scenario 4) a more ancient (between 10^3^ and 5 × 10^5^ generations in the past) expansion, (Scenario 5) an expansion followed by a decrease (*N*_e_ *&gt; N*_0_,*N*_1_), (Scenario 6) a bottleneck (*N*_b_ *<N*_0_ *,N*_1_), and (Scenario 7) a constant effective population size (*N*_0_ = *N*_1_). The parameters *t*_1_ (*t*_1_ < 500 generations i.e., ≈5,000 years) and *t*_2_ (*t*_2_ > 500 generations) were chosen to reflect relatively recent events that may be linked to anthropogenic factors, or more ancient events, such as glacial/interglacial transitions. For Scenarios 5 and 6, the end of the expansion/bottleneck was set at five generations in the past, which approximately corresponds to the ban on commercial exploitation of the tiger shark in Reunion Island (in 1999). Generation time was supposed to be around 7–10 years (Branstetter, Musick, & Colvocoresses, 1987; Holmes et al., 2015; Kneebone, Natanson, Andrews, & Howell, 2008; Wintner & Dudley, 2000).

To run this analysis, we considered all the individuals from Reunion Island, as it was the location with the highest number of individuals and may be the most representative of the genetic diversity of the whole population. It has indeed been shown that pooling individuals from different sampling locations, even with nonsignificant pairwise differentiation values may bias results (Lombaert et al., 2014). Nevertheless, the analysis was also run using only samples from New Caledonia (*n* = 23), to consolidate the results.

For each scenario, 10^6^ simulated datasets were run. To select the best scenario, posterior probabilities were computed via logistic regression on the 1% of simulated datasets closest to the empirical data (Cornuet et al., 2008). Summary statistics were transformed by linear discrimination analysis prior to logistic regression to reduce correlation among explanatory variables and provide conservative estimates of scenario discrimination (Estoup et al., 2012). Posterior distributions of all parameters were then estimated from the selected model, based on the 1% of simulated datasets closest to the empirical data. More details on the ABC analysis are provided in Appendix S3.

## 3 RESULTS

### 3.1 Genetic diversity analyses

#### 3.1.1 27‐msat dataset

Null alleles were detected for several loci in several sampling locations but were not constant among locations and were not correlated with significant deviations from HWE. All loci were thus kept for further analyses. Global significant LD was detected for 21 tests over 2,457 after FDR correction (0.85%, *p* < 0.05) only, and all loci were thus considered independent. The mean rarified allelic richness (±standard error [*SE*]) was relatively constant among locations, varying from 2.86 ± 0.30 in AUS1 (Western Australia) to 3.05 ± 0.28 in New Caledonia while *H*_E_ varied from 0.52 ± 0.06 in Reunion Island to 0.60 ± 0.05 in Zanzibar and *H*_O_ from 0.49 ± 0.05 in South Africa to 0.60 ± 0.06 in Zanzibar (Table 2). No significant deviation from HWE was revealed for any location. The mean rarified private allelic richness varied from 0.11 ± 0.04 in AUS1 (Western Australia) to 0.19 ± 0.05 in New Caledonia (Table 2) and was relatively constant among locations.

**Table 2:** Summary statistics for each sampling location (AUS1, Western Australian coast; AUS2, Queensland, Australia; AUS3, New South Wales, Australia; AUS4, Northern Territories, Australia; BRA, Brazil; COR, Coral Sea; HAW, Hawaii; NCA, New Caledonia; RUN, Reunion Island; SEY, Seychelles; SAF, South Africa; ZAN, Zanzibar) for the 27‐msat and 8‐msat datasets

	*N*	*A*_r_	*A*_rp_	*H*_O_	*H*_E_	*F*_IS_
27 loci						
ZAN	8	3.01 (0.33)	0.14 (0.05)	0.60 (0.06)	0.60 (0.05)	0.01
SEY	24	2.96 (0.31)	0.14 (0.04)	0.57 (0.05)	0.57 (0.05)	0.01
RUN	167	3.02 (0.28)	0.19 (0.04)	0.50 (0.05)	0.52 (0.06)	0.05
SAF	34	3.02 (0.30)	0.16 (0.04)	0.49 (0.05)	0.55 (0.05)	0.12
AUS1	9	2.86 (0.30)	0.11 (0.04)	0.55 (0.05)	0.58 (0.05)	0.05
AUS2	10	2.99 (0.30)	0.18 (0.06)	0.53 (0.05)	0.59 (0.05)	0.1
NCA	23	3.05 (0.28)	0.18 (0.03)	0.53 (0.06)	0.58 (0.05)	0.09
8 loci						
ZAN	8	5.75 (1.10)	0.25 (0.15)	0.61 (0.12)	0.67 (0.11)	0.08
SEY	24	5.66 (1.13)	0.17 (0.12)	0.65 (0.06)	0.69 (0.05)	0.06
SAF	34	5.43 (1.02)	0.11 (0.06)	0.59 (0.04)	0.66 (0.04)	0.07
RUN	167	5.79 (1.04)	0.11 (0.04)	0.65 (0.02)	0.69 (0.02)	0.04
AUS1	56	5.76 (1.02)	0.11 (0.04)	0.68 (0.03)	0.70 (0.03)	0.01
AUS4	62	5.81 (1.06)	0.18 (0.08)	0.71 (0.03)	0.70 (0.03)	−0.04
COR	37	5.89 (1.11)	0.08 (0.05)	0.64 (0.04)	0.68 (0.04)	0.04
AUS2	74	5.80 (1.03)	0.08 (0.03)	0.68 (0.03)	0.70 (0.03)	0.01
AUS3	81	5.80 (1.08)	0.10 (0.07)	0.70 (0.03)	0.69 (0.03)	−0.03
NCA	34	5.53 (0.97)	0.07 (0.04)	0.62 (0.04)	0.67 (0.04)	0.06
HAW	21	5.85 (1.12)	0.10 (0.07)	0.67 (0.06)	0.68 (0.06)	0.01
BRA	8	4.63 (0.73)	0.49 (0.17)	0.73 (0.07)	0.72 (0.07)	−0.16

Populations are ordered along a west to east and north to south gradient beginning at locations from the eastern African coast.

#### 3.1.2 8‐msat dataset

The mean rarified allelic richness varied from 4.63 ± 0.73 in Brazil to 5.89 ± 1.11 in Coral Sea while *H*_E_ and *H*_O_ varied from 0.66 ± 0.04 in South Africa to 0.72 ± 0.07 in Brazil and from 0.59 ± 0.04 in South Africa to 0.73 ± 0.07 in Brazil, respectively (Table 2). No significant deviations from HWE were revealed. The mean rarified private allelic richness was rather constant among locations but was higher for Brazil, varying from 0.07 ± 0.04 in New Caledonia to 0.49 ± 0.17 in Brazil (Table 2).

#### 3.1.3 Mitochondrial diversity indices for the three mitochondrial genes

We obtained sequences of 869 bp for *CR*, 652 pb for *COI* and 931 bp for *cytb*.

Using only our samples, the three genes resolved 9, 4, 14 haplotypes with 7, 3, and 12 polymorphic sites, respectively. Total haplotype diversity (*h*) was lower for *COI* (0.09 ± 00) than for *cytb* (0.47 ± 0.00) and *CR* (0.48 ± 0.00). Similar results were observed for the total nucleotide diversity (π), varying from 0.00014 ± 0.00000 for *COI* to 0.00063 ± 0.00001 and 0.00068 ± 0.00000 for *cytb* and *CR*, respectively. Overall, variations of haplotype and nucleotide diversities were not constant across locations and across genes, with lowest values estimated for Reunion Island (*h* = 0.34 ± 0.01 and π = 0.00047 ± 0.00005) and Zanzibar (*h* = 0.00 ± 0.00 and π = 0.00000 ± 0.00000) and highest values for AUS1 (Western Australia; *h* = 0.78 ± 0.44 and π = 0.00115 ± 0.00032) for *CR* and New Caledonia (*h* = 0.61 ± 0.03 and π = 0.00099 ± 0.00018) for *cytb*. For *COI*, nearly all locations showed null haplotype and nucleotide diversities, except for South Africa (*h* = 0.07 ± 0.01 and π = 0.00010 ± 0.00004), New Caledonia (*h* = 0.09 ± 0.02 and π = 0.00013 ± 0.00006), and Reunion Island (*h* = 0.21 ± 0.01 and π = 0.00032 ± 0.00007; Appendix S4).

**Figure 3:**
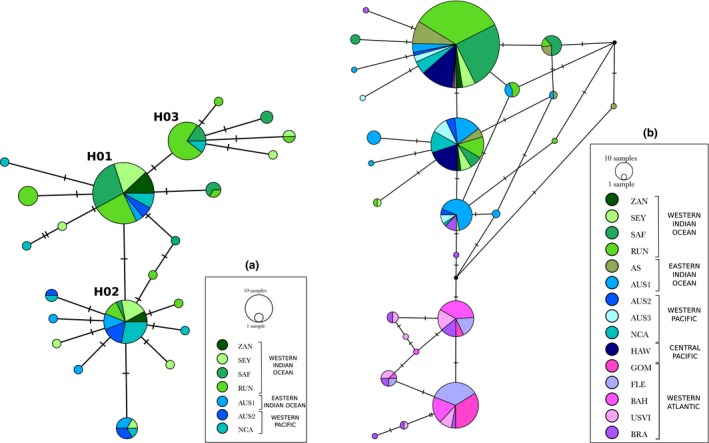
TCS statistical parsimony networks for the tiger shark *Galeocerdo cuvier* constructed with (a) the *CR‐COI‐cytb* dataset (22 haplotypes) and (b) the *CR* dataset (25 haplotypes). Each circle represents a haplotype and each trait, a mutation. Circle size is proportional to the number of individuals harboring each haplotype and colors correspond to sampling locations (AS: Andaman Sea; AUS1: Western Australian coast; AUS2: Queensland, Australia; AUS3: New South Wales, Australia; AUS4: Northern Territories, Australia; BAH: Bahamas; COR; BRA: Brazil; Coral Sea; FLE: Florida East Coast; HAW: Hawaii; GOM: Gulf of Mexico; NCA: New Caledonia; RUN: Reunion Island; SEY: Seychelles; SAF: South Africa;USVI:US Virgin Islands; ZAN: Zanzibar)

The *CR‐COI‐cytb* dataset (2,452 bp; *N*_S_ = 127) resolved 22 haplotypes and 22 polymorphic sites, with an overall haplotype diversity of 0.78 ± 0.00 and a nucleotide diversity of 0.00053 ± 0.00000 (Table 3a). No partitioning schemes were detected within the *CR‐COI‐cytb* sequence, and the HKY85 model of substitution was selected. Bayesian analysis on the *CR‐COI‐cytb* dataset showed good convergence and mixing, with ESS above 200 (Appendix S5). Nevertheless, no lineages were strongly supported as no internal nodes showed high support. This was retrieved on the TCS statistical parsimony network built from the *CR‐COI‐cytb* dataset, with all haplotypes separated by only one or two mutations, and no clear geographic structuring (Figure 3a; Appendix S6). Three main haplotypes were identified, represented by 50, 25, and 9 individuals from distinct locations.

**Table 3:** Summary statistics for each sampling location of *Galeocerdo cuvier* for (a) the 2,452 bp *CR‐COI‐cytb* dataset and (b) the *CR* dataset: *N*_S_, number of individuals sequenced in this study; *N*_Bernard_, number of individuals sequenced in Bernard et al. (2016); *N*_S+Bernard_, total number of individuals sequenced; *H*, number of haplotypes, *h*, haplotype diversity; *S*, number of polymorphic sites; π, nucleotide diversity

(a) *CR_COI_cytb*		*N*_S_		*H*	*S*	*h*	π
ZAN		8		2	1	0.43 (0.06)	0.00018 (0.00007)
SEY		20		8	8	0.76 (0.02)	0.00053 (0.00009)
SAF		23		6	5	0.62 (0.02)	0.00035 (0.00006)
RUN		39		9	8	0.77 (0.01)	0.00049 (0.00006)
AUS1		9		5	4	0.86 (0.03)	0.00050 (0.00013)
AUS2		10		4	3	0.78 (0.03)	0.00042 (0.00011)
NCA		18		8	9	0.82 (0.02)	0.00062 (0.00010)
Total		127		22	22	0.78 (0.00)	0.00053 (0.00000)

(b) *CR*	*N*_Bernard_	*N*_S_	*N*_S+Bernard_	*H*	*S*	*h*	π
ZAN	–	8	8	2	1	0.43 (0.06)	0.00049 (0.00020)
SEY	–	20	20	4	3	0.57 (0.02)	0.00081 (0.00016)
SAF	56	24	80	4	3	0.39 (0.01)	0.00048 (0.00005)
RUN	–	103	103	6	5	0.34 (0.01)	0.00047 (0.00005)
AS	31	–	31	5	4	0.52 (0.02)	0.00084 (0.00013)
AUS1	49	9	58	9	6	0.78 (0.00)	0.00138 (0.00013)
AUS2	–	10	10	3	2	0.69 (0.03)	0.00095 (0.00026)
AUS3	19	–	19	4	3	0.68 (0.02)	0.00101 (0.00019)
NCA	–	25	25	3	2	0.56 (0.01)	0.00069 (0.00013)
HAW	48	–	48	2	1	0.48 (0.01)	0.00055 (0.00008)
GOM	26	–	26	2	1	0.21 (0.02)	0.00024 (0.00007)
FLE	35	–	35	4	7	0.49 (0.01)	0.00091 (0.00013)
BAH	33	–	33	5	7	0.60 (0.01)	0.00106 (0.00015)
USVI	22	–	22	7	9	0.80 (0.01)	0.00196 (0.00028)
BRA	20	–	20	9	11	0.87 (0.01)	0.00317 (0.00044)
Total	339	199	538	25	16	0.74 (0.00)	0.00280 (0.00000)

AS: Andaman Sea; AUS1: Western Australian coast; AUS2: Queensland: Australia; AUS3: New South Wales: Australia; BAH: Bahamas; BRA: Brazil**;** FLE: Florida East Coast; GOM: Gulf of Mexico; HAW: Hawaii; NCA: New Caledonia; RUN: Reunion Island; SAF: South Africa; SEY: Seychelles; USVI: US Virgin Islands; ZAN: Zanzibar.

#### 3.1.4 Mitochondrial diversity on the CR dataset

Within the *CR* dataset, we analyzed 538 *CR* sequences and resolved 25 haplotypes and 16 polymorphic sites, with an overall haplotype diversity *h* of 0.74 ± 0.00 and a nucleotide diversity π of 0.00280 ± 0.00000 (Table 3b). We retrieved only two new haplotypes compared to the study of Bernard et al. (2016), represented by three individuals (from the western Indian Ocean). From locations in common in both studies, South Africa and AUS1 (Western Australia), *h* and π for the *CR* did not vary when adding individuals from Bernard et al. (2016) (Table 3b; Appendix S4). As shown by the TCS network built, samples collected for this study presented the three main haplotypes identified by Bernard et al. (2016) in the Indian and Pacific Oceans and none of the haplotypes identified in the western Atlantic (Figure 3b).

### 3.2 Population genetic structure

**Figure 4:**
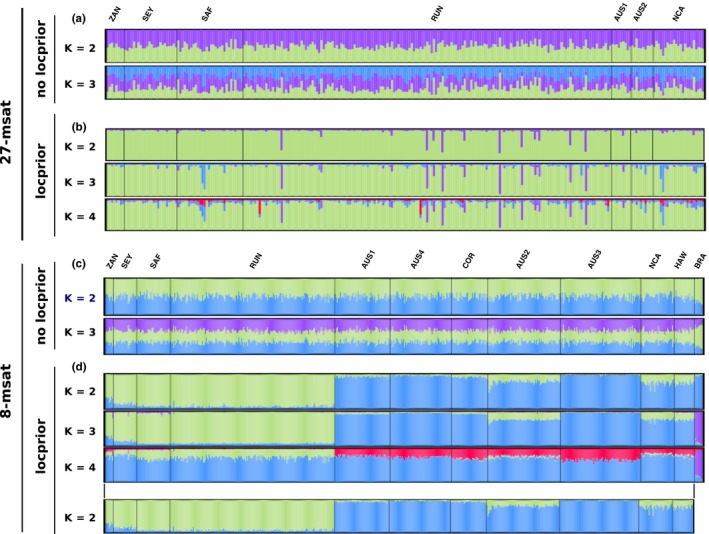
*Galeocerdo cuvier* assignment probabilities of individuals to putative clusters assuming correlated allele frequencies and admixture as performed by STRUCTURE. (a) Average probability of membership (*y* axis) of individuals (*N* = 275, *x *axis) for major modes of *K* varying from 2 to 3, with the 27‐msat dataset and no *a priori* sampling location information. (b) Average probability of membership (*y* axis) of individuals (*N* = 275, *x *axis) for major modes of *K* varying from 2 to 3, with the 27‐msat dataset and the LOCPRIOR model. (c) Average probability of membership (*y* axis) of individuals (*N* = 606, *x *axis) for major modes of *K* varying from 2 to 3, with the 8‐msat dataset and no *a priori* sampling location information. (d) Average probability of membership (*y* axis) of individuals (*N* = 606, *x *axis) for major modes of *K* varying from 2 to 4, with the 8‐msat dataset and the LOCPRIOR model. AUS1, Western Australian coast; AUS2, Queensland, Australia; AUS3, New South Wales, Australia; AUS4, Northern Territories, Australia; BRA, Brazil; COR, Coral Sea; HAW, Hawaii; NCA, New Caledonia; RUN, Reunion Island; SEY, Seychelles; SAF, South Africa; ZAN, Zanzibar

Performing Bayesian clustering analyses without the LOCPRIOR model, no distinct genetic clusters were retrieved in either the 27‐msat or the 8‐msat datasets (Figure 4a, c).

Using the LOCPRIOR model on these both microsatellite datasets, a single mode regrouping the five runs was retrieved for each *K*. For the 8‐msat dataset, the highest averaged log likelihood of observing the data (‐15,986.26 ± 11.59) was retrieved for *K* = 3. Individuals from the western Indian Ocean (ZAN, SEY, SAF, and RUN) belonged to one genetic cluster, those from the eastern Indian Ocean (AUS1), the western (AUS2, AUS3, AUS4, COR, and NCA), and Central (HAW) Pacific to a second one, and those from the western Atlantic (BRA) to a third one (Figure 4d). Nevertheless, with the 27‐msat dataset, the two clusters previously identified did not appear (Figure 4b). Indeed, while *K* = 4 presented the highest likelihood of observing the data (‐16,235.32 ± 66.01), from *K* = 2 each new cluster was mostly uninformative, with individual membership proportions in new clusters extremely low (respectively, 3.43% ± 0.82%, 4.13% ± 0.74%, and 2.00% ± 0.45% for *K* = 2, *K* = 3, and *K* = 4).

**Figure 5:**
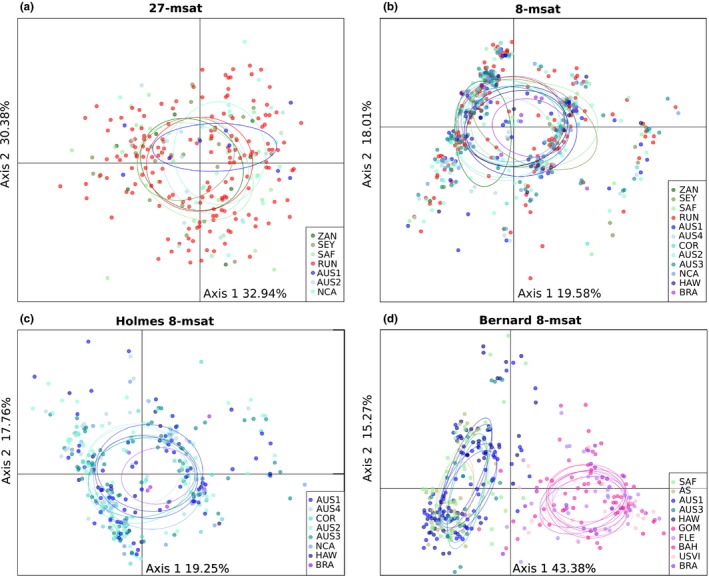
*Galeocerdo cuvier* scatterplot output from DAPC analyses performed using the four microsatellite datasets, and using the first and second components (a) 27‐msat dataset, (b) 8‐msat dataset, (c) Holmes 8‐msat dataset, (d) Bernard 8‐msat dataset. Dots represent individuals colored by their sampling location (AS: Andaman Sea; AUS1: Western Australian coast; AUS2: Queensland, Australia; AUS3: New South Wales, Australia; AUS4: Northern Territories, Australia; BAH: Bahamas; BRA: Brazil; COR: Coral Sea; HAW: Hawaii; GOM: Gulf of Mexico; FLE: Florida East Coast; NCA: New Caledonia; RUN: Reunion Island; SEY: Seychelles; SAF: South Africa; USVI:US Virgin Islands; ZAN: Zanzibar)

Discriminant analyses of principal components performed on both 27‐msat and 8‐msat datasets did not retrieve distinct genetic clusters, with 95% confidence ellipses for each location overlapping (Figure 5a, b; BIC in Appendix S7). For the analysis on the 27‐msat dataset, the first axis explained 32.94% and the second 30.38% of total inertia (Figure 5a) and no clear groups were identified. For the 8‐msat dataset, when assigning individuals to five clusters (i.e., defining *K* = 5), the first axis explained 19.58% of total inertia and the second axis 18.01% and ellipses for each location overlapped, with no clear clusters identified (Figure 5b). Nevertheless, with the same dataset, for lower values of *K* (3 and 4), individuals seemed to cluster into three groups, not related to their geographical origins (Appendix S8a). As DAPC analyses were not performed in the studies of Holmes et al. (2017) and Bernard et al. (2016), we performed them on both Holmes 8‐msat and Bernard 8‐msat datasets, to compare the results obtained (Figure 5c, d, BIC in Appendix S7). Using Holmes 8‐msat dataset, we observed the same patterns with the 8‐msat dataset (i.e., three clusters not related to the individual geographical origin for *K* varying from 2 to 4, and for higher *K*, no clustering pattern). With the Bernard 8‐msat dataset, individuals were clearly clustered into two groups, one from the western Atlantic and one from the Indo‐Pacific (Figure 5d). The three clusters identified with the 8‐msat and Holmes 8‐msat datasets were not observed with the Bernard 8‐msat dataset for any *K*.

In conclusion, for both 27‐msat and 8‐msat datasets, neither the structure analysis without the LOCPRIOR model nor the DAPC highlighted clear genetic clusters, even among locations within the Indo‐Pacific nor within the western Atlantic. Only the structure analysis with the LOCPRIOR model succeeded to identify different genetic clusters in the Indo‐Pacific, but only using the 8‐msat dataset. These clusters were not identified in any of the other analyses, including the same analysis with the 27‐msat dataset, which, if this pattern clearly exists, should have attributed individuals into two different genetic clusters. Thus, as no clear geographic structuring was retrieved using these two microsatellite datasets, we did not perform analyses of molecular variances, and instead directly calculated pairwise differentiation estimates.

Concerning the 27‐msat dataset, no pairwise *F*_ST_ values were found to be significantly different from 0 (*F*_ST_ = [0.000, 0.012], all *p* > 0.05 after FDR correction; Table 4a). Nevertheless, weak but significant *D*_est_ values were retrieved between New Caledonia and all other locations (*D*_est_ = [0.030, 0.052], all *p* < 0.05 after FDR correction), except Zanzibar (*D*_est_ = 0.026^NS^). AUS2 (Queensland, Australia) was also significantly different from the Seychelles, South Africa and Reunion Island (*D*_est_ = [0.034, 0.044], all *p* < 0.05 after FDR correction) and a low but significant value was also retrieved between Reunion Island and South Africa (*D*_est_ = 0.014, *p* < 0.05 after FDR correction; Table 4a).

**Table 4:** Genetic differentiation between *Galeocerdo cuvier* sampling locations estimated with Weir and Cockerham's *F*_ST_ (lower‐left matrix) and Jost's *D*_est_. (upper‐right matrix) for (a) the 27‐msat dataset, and (b) the 8‐msat dataset

(a)	ZAN	SEY	SAF	RUN	AUS1	AUS2	NCA
ZAN (8)	–	0.000	0.000	0.000	0.000	0.019	0.026
SEY (24)	0.001	–	0.007	0.003	0.005	0.044*	0.047**
SAF (34)	0.000	0.002	–	0.014*	0.028	0.038*	0.052**
RUN (167)	0.000	0.000	0.006	–	0.014	0.034*	0.034**
AUS1 (9)	0.000	0.008	0.012	0.012	–	0.022	0.030*
AUS2 (10)	0.000	0.004	0.000	0.000	0.000	–	0.040*
NCA (23)	0.000	0.008	0.008	0.001	0.004	0.000	–

(b)	ZAN	SEY	SAF	RUN	AUS1	AUS4	COR	AUS2	AUS3	NCA	HAW	BRA
ZAN (8)	–	0.000	0.000	0.000	0.000	0.000	0.000	0.000	0.000	0.000	0.000	0.331**
SEY (24)	0.002	–	0.035	0.004	0.000	0.014	0.003	0.003	0.027	0.025	0.000	0.376**
SAF (34)	0.008	0.007	–	0.032*	0.056**	0.032	0.036	0.025	0.039*	0.033	0.033	0.342**
RUN (167)	0.000	0.003	0.010*	–	0.019*	0.017*	0.006	0.006	0.018**	0.017	0.005	0.377**
AUS1 (56)	0.002	0.000	0.011*	0.004	–	0.010	0.000	0.021	0.021*	0.019	0.000	0.366**
AUS4 (62)	0.000	0.007	0.009*	0.003	0.001	–	0	0.000	0.000	0.011	0.002	0.355**
COR (37)	0.001	0.007	0.011	0.000	0.000	0.001	–	0.000	0.000	0.008	0.019	0.406**
AUS2 (74)	0.000	0.004	0.008	0.000	0.003	0.000	0.000	–	0.000	0.013	0.017	0.355**
AUS3 (81)	0.000	0.007	0.009*	0.002	0.003	0.000	0.000	0.000	–	0.018	0.000	0.365**
NCA (34)	0.003	0.007	0.008	0.002	0.002	0.004	0.001	0.004	0.003	–	0.002	0.367**
HAW (21)	0.000	0.004	0.006	0.001	0.000	0.000	0.001	0.001	0.000	0.000	–	0.323**
BRA (8)	0.039	0.048*	0.033*	0.047**	0.037*	0.046**	0.051**	0.044**	0.038**	0.056**	0.030*	–

Tests significance were assessed after FDR correction and values significantly different from zero are indicated in bold; *P < 0.05; **P < 0.01. In parentheses are indicated the number of individuals used for analyses. AUS1: Western Australian coast; AUS2: Queensland: Australia; AUS3: New South Wales: Australia; AUS4: Northern Territories: Australia; BRA: Brazil; COR: Coral Sea; HAW: Hawaii; NCA: New Caledonia; RUN: Reunion Island; SAF: South Africa; SEY: Seychelles; ZAN: Zanzibar.

Considering the 8‐msat dataset, significant pairwise *F*_ST_ values were only found between: Brazil and all other locations (*F*_ST_ = [0.030, 0.056], all *p* < 0.05 after FDR correction; Table 4b), except Zanzibar (*F*_ST_ = 0.039^NS^); and between South Africa and Reunion Island, AUS1 (West Australia), AUS4 (North Australia) and AUS3 (East Australia) (*F*_ST_ = [0.009, 0.011], all *p* < 0.05 after FDR correction). Significant *D*_est_ values were also retrieved between: Brazil and all other locations (*D*_est_ = [0.323, 0.406], all *p* < 0.01 after FDR correction); between Reunion Island and South Africa, AUS1 (West Australia) and AUS3 (East Australia) (*D*_est_ = [0.032, 0.056], all *p* < 0.05 after FDR correction); between Reunion Island and AUS1, AUS4, and AUS3 (*D*_est_ = [0.017, 0.019], all *p* < 0.05 after FDR correction); and between AUS1 and AUS3 (*D*_est_ = 0.021, *p* < 0.05; Table 4b).

Pairwise *Φ*_ST_ values for the *CR‐COI‐cytb* dataset were highly significant between: Reunion Island and AUS1 (West Australia); between AUS2 (East Australia) and New Caledonia; as well as between South Africa and AUS1 (West Australia), AUS2 (East Australia), and New Caledonia (*Φ*_ST_ = [0.165, 0.387], all *p* < 0.001 after FDR correction; Table 5). Values were lower but also significant between the Seychelles and South Africa and between the Seychelles and Reunion Island (Φ_ST_ = 0.104 and Φ_ST_ = 0.110, all *p* < 0.01 after FDR correction; Table 5). When using the *CR* dataset, highly significant values were observed between locations of the northwestern Atlantic Ocean (i.e., GOM, FLE, BAH and USVI) and locations from the Indian and Pacific Oceans (Φ_ST_ = [0.697, 0.954], all *p* < 0.001 after FDR correction; Table 5), and also between Brazil and locations from the Indian and Pacific Oceans (Φ_ST_ = [0.485, 0.793], all *p* < 0.001 after FDR correction). Within the Indo‐Pacific and the western Atlantic, some locations were significantly differentiated from each other, but this was not related to geographical distance or separation by coastline (see Table 5).

**Table 5:** Genetic differentiation between *Galeocerdo cuvier* sampling locations estimated with Weir and Cockerham's Φ_ST_ for the *CR‐COI‐cytb* dataset (upper‐right matrix; number of individuals indicated in parentheses on the first line) and for the *CR* dataset (lower‐left matrix; number of individuals indicated in parentheses on the first column)

	ZAN (8)	SEY (20)	SAF (23)	RUN (39)	AS	AUS1 (9)	AUS2 (10)	AUS3	NCA (18)	HAW	GOM	FLE	BAH	USVI	BRA
ZAN (8)	–	0.000	0.054	0.100		0.228	0.179		0.016						
SEY (20)	0.000	–	**0.104** *	**0.110** *		0.084	0.043		0.000						
SAF (80)	0.021	**0.174** ***	–	0.033		**0.387** ***	**0.346** ***		**0.183** ***						
RUN (103)	0.000	**0.114** *	**0.020** *	–		**0.327** ***	**0.290** ***		**0.165** **						
AS (31)	0.000	0.056	0.014	0.029	–										
AUS1 (58)	**0.280** **	**0.215** *	**0.486** ***	**0.463** ***	**0.339** ***	–	0.000		0.010						
AUS2 (10)	0.207	0.074	**0.467** ***	**0.418** ***	**0.258** **	0.015	–		0.000						
AUS3 (19)	0.124	0.037	**0.388** ***	**0.340** ***	**0.206** **	**0.073** *	0.000	–							
NCA (25)	0.048	0.000	**0.290** ***	**0.236** ***	**0.140** **	**0.182** ***	0.017	0.000	–						
HAW (48)	0.000	0.000	**0.165** ***	**0.111** ***	**0.073** *	**0.300** ***	**0.175** *	**0.109** *	0.013	–					
GOM (26)	**0.954** ***	**0.922** ***	**0.937** ***	**0.936** ***	**0.911** ***	**0.818** ***	**0.923** ***	**0.904** ***	**0.925** ***	**0.931** ***	–				
FLE (35)	**0.869** ***	**0.857** ***	**0.903** ***	**0.907** ***	**0.857** ***	**0.774** ***	**0.835** ***	**0.835** ***	**0.862** ***	**0.885** ***	0.016	–			
BAH (33)	**0.841** ***	**0.832** ***	**0.890** ***	**0.895** ***	**0.834** ***	**0.745** ***	**0.801** ***	**0.806** ***	**0.838** ***	**0.867** ***	**0.245** ***	**0.082** *	–		
USVI (22)	**0.752** ***	**0.764** ***	**0.865** ***	**0.874** ***	**0.780** ***	**0.697** ***	**0.705** ***	**0.731** ***	**0.778** ***	**0.829** ***	**0.195** ***	**0.067** *	0.000	–	
BRA (20)	**0.564** ***	**0.598** ***	**0.777** ***	**0.793** ***	**0.640** ***	**0.518** ***	**0.485** ***	**0.542** ***	**0.616** ***	**0.707** ***	**0.327** ***	**0.222** ***	**0.128** ***	0.059	–

AS: Andaman Sea; AUS1: Western Australian coast; AUS2: Queensland: Australia; AUS3: New South Wales: Australia; BAH: Bahamas; BRA: Brazil; FLE: Florida East Coast; GOM: Gulf of Mexico; HAW: Hawaii; NCA: New Caledonia; RUN: Reunion Island; SAF: South Africa; SEY: Seychelles; USVI: US Virgin Islands; ZAN: Zanzibar. Tests significance were assessed after FDR correction and values significantly different from zero are indicated in bold.

*P < 0.05; **P < 0.01; ***P < 0.001.

### 3.3 Population demography

Considering the *CR‐COI‐cytb* dataset and all locations separately, no evidence of historical population expansions or contractions was found when estimating Tajima's *D* (all *p* > 0.05; Appendix S9). Nevertheless, pooling all locations, a significantly negative *D* value was found (*D* = −1.83, *p* < 0.01), suggesting a population expansion. Furthermore, all Fu's *F*_S_ estimates were significantly negative, either considering all locations separately (except for Zanzibar and Western Australia AUS2) or pooling all locations (*F*_S_ = [−18.12, −1.99]; all *p* < 0.05; Appendix S9), also indicative of a population expansion.

Concerning the ABC analyses (Appendix S10), observed summary statistics for all scenarios fell within the distribution of simulated summary statistics, suggesting adequate choice of prior distributions (Appendix S10a). Scenario 6 (population decrease at time *t* from *N*_1_ to *N*_b_, followed by an expansion from *N*_b_ to *N*_0_ five generations ago; Figure 2) presented the highest posterior probability based both on the logistic regression‐based estimates and the direct estimate of posterior probability (Appendices S10b and S10c). Other scenarios received no statistical support. Furthermore, posterior error rates were relatively low, with values of 0.342 and 0.294 using the direct and logistic approaches, respectively. We thus estimated parameter values using data simulated under Scenario 6 (Table 5). The population size decrease during the bottleneck event (*N*_b_ *)* was equal to 111 (95% confidence interval [CI] = [43, 369] and root mean square error [RMSE] = 0.958), the ancestral effective population sizes (*N*_1_) equal to 5,150 (95% CI = [1,120, 9,710], RMSE = 1.049) and the time at which the bottleneck began (*t*) estimated to 319 (95% CI = [65, 913]) generations, corresponding to approximately 2,000 – 3,000 years (RMSE = 1.347). However, the actual effective population size *N*_0_ was less precisely inferred (Table 6; Appendix S11).

**Table 6:** Characteristics of demographic parameter posterior distributions estimated using *Galeocerdo cuvier* individuals from Reunion Island with diyabc under Scenario 6 (population decrease at time *t* from *N*_1_ to *N*_b_, followed by an expansion from *N*_b_ to *N*_0_ five generations ago)

Parameter	Median	2.5% quantile	97.5% quantile	RMSE
*N*_b_	111	43	369	0.958
*t*	319	65	913	1.347
*N*_0_	5760	639	9,820	2.839
*N*_1_	5,150	1,120	9,710	1.049

*N*_0_: the actual effective population size; *N*_1_: the ancestral effective population size; *N*_b_: the effective population size during a bottleneck; *t*: beginning of the bottleneck period; RMSE: root mean square error.

Similar results were found when performing the analysis including only individuals from New Caledonia (Appendices S12, S13, and S14).

## 4 DISCUSSION

Ideally, every population genetic study needs the greatest sampling coverage and the greatest number of microsatellite loci (Koskinen, Hirvonen, Landry, & Primmer, 2004; Meirmans, 2015). Nevertheless, achieving an extensive sampling is difficult, especially for elusive species, while elaborating numerous molecular markers remains expensive. Thus, researchers continually strive to find a balance between both strategies but ultimately have to settle for what they can afford. Hence, some datasets encompass a great sampling coverage while others strive for a greater number of markers. In this study, we combined datasets to improve both sample sizes and number of markers to improve the power of analyses of tiger shark population structure. Comparing results from the datasets, they were not always congruent: the dataset with the greatest number of samples but the lowest number of molecular markers highlighted potential differentiation between the western Indian Ocean and the eastern Indian Ocean/Pacific, which was not identified with the dataset with larger number of markers but fewer samples. It demonstrates how difficult it may be to interpret results from multiple studies with different population samples and molecular screening effort.

This study investigated the population structure and demographics of the tiger shark by adding additional samples and new molecular markers to recent studies (Bernard et al., 2016; Holmes et al., 2017). Compared to these previous studies, we carried out intensive sampling in the western Indian Ocean, a region in which only one location had previously been sampled (South Africa). We also used a higher number of microsatellite and mitochondrial loci in our analyses, thus expanding the picture of tiger shark population structure. The results confirmed here the genetic differentiation between the populations from the Indo‐Pacific and the western Atlantic, with both microsatellite and mitochondrial markers, while an important genetic connectivity was detected within and between the Indian and the Pacific Oceans. Furthermore, we investigated for the first time variations in effective population size at the scale of the Indo‐Pacific and highlighted the probable occurrence of a bottleneck 2,000‐3,000 years ago.

### 4.1 Connectivity between the western Atlantic and the Indo‐Pacific

Genetic differentiation between tiger sharks from the western Atlantic and the Indo‐Pacific could only be investigated with the 8‐msat and *CR* datasets as we did not obtain samples from the western Atlantic. By adding new sampling sites from the western Indian Ocean, the present study confirmed a genetic differentiation between the two regions. Both structure (using the LOCPRIOR model and at *K* = 3, i.e., not the first level of differentiation) and mitochondrial haplotype network clustered individuals from Brazil (and more largely the western Atlantic when analyzing individuals from Bernard et al. (2016)) separately from the individuals sampled from the Indo‐Pacific. It is possible that the low number of markers used created a false differentiation; however, this was contradicted by some of the other results. Indeed, pairwise differentiation indices were higher for comparisons between localities in the Indo‐Pacific and the Atlantic compared to comparisons within the Indo‐Pacific, both in the current study and in Bernard et al. (2016) and Holmes et al. (2017). Furthermore, the TCS haplotype networks constructed by Bernard et al. (2016) with the mitochondrial markers *CR* and *COI* clearly showed that no haplotypes were shared between the Indo‐Pacific and the northwestern Atlantic, and only one was shared between the Indo‐Pacific and Brazil, suggesting a real differentiation.

### 4.2 Population connectivity within the Indo‐Pacific

Patterns of genetic differentiation within the Indo‐Pacific were more difficult to interpret. First, considering the 8‐mast dataset, structure with the LOCPRIOR model identified two genetic clusters, one grouping individuals from the western Indian Ocean (i.e., ZAN, SEY, SAF, and RUN) and the other, locations from the eastern Indian Ocean (Australia) and from the Pacific (i.e., AUS1, AUS2, AUS3, AUS4, COR, NCA, and HAW). However, structure without the LOCPRIOR model and DAPC did not partition the individuals into distinct clusters. Considering the 27‐msat dataset, none of the analyses performed identified distinct clusters within the Indo‐Pacific. Increasing the number of markers has improved the resolution, highlighting the need of sufficient number of microsatellite loci for population genetics analyses (Meirmans, 2015; Putman & Carbone, 2014). Thus, it is likely that the clustering pattern identified by structure (LOCPRIOR model) with the 8‐msat dataset does not reflect a biological pattern, but instead the microsatellite characteristics when used in low number.

Microsatellite differentiation estimates between population pairs from the Indo‐Pacific were dependent on the differentiation index used (*F*_ST_ or *D*_est_) and were not related to whether locations were connected by coastlines or separated by large oceanic expanses. If we only take into account differentiation values significantly different from zero for both indices (*F*_ST_ and *D*_est_), genetic differentiation was only highlighted for the 8‐msat dataset and not particularly between very distant locations: South Africa and Reunion Island (not identified with the 27‐msat dataset despite similar numbers of individuals in both datasets), South Africa and Western Australia (AUS1) or South Africa and the northeastern Australia (AUS3). When using the 27‐msat dataset, none of the population pairs were significantly differentiated, supporting the hypothesis of one genetic cluster in the Indo‐Pacific. The same pattern was established by Bernard et al. (2016), except for Hawaii that was significantly differentiated from all other locations. This discrepancy concerning Hawaii could be due to the lower sample size from eastern Australia (21 samples) used by Bernard et al. (2016) to assess *F*_ST_ and *D*_est_ and to the structure analysis.

The TCS haplotype networks constructed in this study and the one of Bernard et al. (2016), using either only the *CR* sequence or the *CR‐COI‐cytb* sequence, showed shared haplotypes among all locations sampled in the Indo‐Pacific. Only three main haplotypes were identified in both studies, separated from one another by one mutation event. This confirms the absence of differentiation among locations from the Indo‐Pacific. Some of the mitochondrial differentiation estimates calculated for both datasets (*CR* or *CR‐COI‐cytb*) were nevertheless significantly different from zero, which would point to some level of genetic differentiation. Notably, it concerned individuals from Reunion Island and South Africa, for which Φ_ST_ estimates were significantly different from individuals from the Seychelles, Australia, and New Caledonia. No significant estimates were calculated between the individuals from Seychelles and those from Australia or New Caledonia, locations separated by almost same distance between South Africa and Australia, which make these results difficult to interpret. It is here useful to remember that Φ_ST_ are based on haplotype frequencies and are highly dependent on sampling.

Thus, altogether, weak genetic differentiation was highlighted between locations in the Indo‐Pacific, both with microsatellite and mitochondrial markers. Tiger sharks are known to cross large oceanic expanses (Ferreira et al., 2015; Hammerschlag et al., 2012; Holmes et al., 2014; Werry et al., 2014), with records of transoceanic migrations in both the Indian (Heithaus et al., 2007) and the Atlantic Oceans (Afonso et al., 2017; Kohler et al., 1998; Kohler & Turner, 2001; Lea et al., 2015). These observations are thus in accordance with the weak genetic differentiation highlighted in the Indo‐Pacific in this and previous studies.

### 4.3 To be or not to be philopatric?

While the evidence remains scant, it is plausible that significant mitochondrial differentiation occurs. From their mitochondrial differentiation estimates, Bernard et al. (2016) inferred matrilineal population structure within the Indo‐Pacific, which they linked to potential female site fidelity (i.e., philopatry) to reproductive areas, probably pupping sites. Female philopatry to nurseries has been hypothesized in many shark species, notably from discordances between mitochondrial and nuclear differentiation estimates (Karl, Castro, Lopez, Charvet, & Burgess, 2011; Pardini et al., 2001; Portnoy et al., 2014; Tillett, Meekan, Field, Thorburn, & Ovenden, 2012), but these discordances may also result from other processes (Prugnolle & de Meeus, 2002). Furthermore, the tiger shark seems to inhabit pelagic waters more frequently than other species such as the lemon shark *Negaprion brevirostris*, for which female philopatry to nurseries has been confirmed (Feldheim et al., 2013). The tiger shark may thus be less constrained in their movements and females may swim to various sites. It is noteworthy that no known tiger shark nurseries have been identified in the Indo‐Pacific, leaving unanswered the hypothesis of female fidelity to coastal pupping areas. The tiger shark is also one of the few species for which multiple paternity (polyandry) has not been identified, although only eight litters in total have been investigated (Holmes et al., 2018; Pirog, Magalon, & Jaquemet, 2019). Yet, this behavior is hypothesized to be linked to philopatry and more structured populations (Chapman, Prodohl, Gelsleichter, Manire, & Shivji, 2004), and the predominance of monoandry in the tiger shark may be another indication pointing to an absence of female philopatry to specific nurseries. All considered further studies, notably identification of nurseries and genotyping of females and juveniles for several years, as well as satellite tracking are needed to fully resolve the occurrence or absence of female tiger shark fidelity to nurseries.

### 4.4 Low genetic diversity and bottleneck

The tiger shark displays moderate genetic diversity with a very low number of mitochondrial haplotypes and haplotype diversity for the sequences studied compared to other shark species, such as the bull shark *Carcharhinus leucas* (Pirog, Jaquemet, et al., 2019), the great white shark *Carcharodon carcharias* (Pardini et al., 2001), the blue shark *Prionace glauca* (Veríssimo et al., 2017), the blacktip reef shark *Carcharhinus melanopterus* (Vignaud et al., 2014), or the tope shark *Galeorhinus galeus* (Chabot, 2015; Chabot & Allen, 2009). Furthermore, using the same protocol on the same date from extraction to marker testing for polymorphism, we characterized 20 microsatellite loci for the bull shark (Pirog et al., 2015), and only eight for the tiger shark (Pirog et al., 2016), which supports a lower genetic diversity in the latter species. Low genetic diversity has also been identified for another pelagic species, the basking shark *Cetorhinus maximus* (Hoelzel, Shivji, Magnussen, & Francis, 2006), for which only six haplotypes were identified using the *CR* marker, over its entire distribution range. This low genetic diversity was thought to be due to the occurrence of a bottleneck during the Holocene (Hoelzel et al., 2006).

Here, the Bayesian analysis, which combined both nuclear and mitochondrial information also provided evidence for the occurrence of a recent bottleneck experienced by the Indo‐Pacific tiger shark population, 2,000–3,000 years ago (during the Holocene). Approximate Bayesian computation has been proven to be one of the most accurate methods to detect recent demographic changes, as evidenced by the study of a large marine mammal for which historic fisheries data were available, the Antarctic fur seal *Arctocephalus gazella* (Hoffman, Grant, Forcada, & Phillips, 2011). Estimates of effective population sizes for the tiger shark remained difficult to infer, but the ancestral size of this population was around 5,000 individuals (95% CI = [1,120, 9,710]) and the bottleneck resulted in an effective population size as low as 111 individuals (95% CI = [43, 369]). This bottleneck may well be responsible for the low genetic diversity presented by the species. Decreases in effective population size within the Holocene have also been identified for several large marine species, notably sea turtles (Molfetti et al., 2013), whales (Baker & Clapham, 2004), and elephant seals (de Bruyn et al., 2009), and also for terrestrial megafauna (Brook & Bowman, 2002). Notably, a similar signal to the one we identified here for the tiger shark was also found in the scalloped hammerhead shark *Sphyrna lewini* in its eastern Pacific range (Nance, Klimley, Galván‐Magaña, Martínez‐Ortíz, & Marko, 2011). Factors responsible for these population declines during the Holocene remain difficult to identify. This period is characterized by a general warming that likely induced population expansions of many marine species (Marko et al., 2010; Uthicke & Benzie, 2003), yet it is unclear how climate changes during this period may have led to population decreases of tropical and subtropical species. A widespread hypothesis for extinctions during the Pleistocene–Holocene is the emergence of diseases (Koutavas, Lynch‐Stieglitz, Marchitto, & Sachs, 2002), notably for corals and sea urchins (Aronson & Precht, 2001; Lessios, 1988). Nevertheless, little is known about diseases and their impacts in sharks, despite an apparently robust immune system (Luer, Walsh, & Bodine, 2004; Walsh et al., 2006). One could also evoke the large volcanic eruptions that took place at this period. The ash layer with the increase of aerosols in the atmosphere absorbed solar radiation, leading to short‐term cooling at regional to global scales, proportional to the magnitude of the eruptive episode (Robock, 2000; Sigl et al., 2015). The subsequent reduction in photosynthesis and the change in species distribution may have led to modifications of trophic networks, possibly impacting marine populations, notably apex predators. It is also possible that prehistoric fisheries were responsible for initiating this decrease, which was then intensified by modern fishing. Indeed, evidence of pelagic fishing was reported as early as 42,000 years ago in the western Pacific (O'Connor, Ono, & Clarkson, 2011). Other studies have provided evidence that prehistoric fishing may have impacted coastal ecosystems in parts of the world (Cooke, 1992; Erlandson & Rick, 2010). Furthermore, the presence of elasmobranchs (sharks, rays, and skates) in prehistoric fisheries is difficult to prove as their cartilaginous skeleton was rarely preserved in archeological sites (Rick, Erlandson, Glassow, & Moss, 2002). Thus, the impacts of prehistoric fishing on these populations may be underestimated. Regardless of the possible causes of tiger shark population decline in the Indo‐Pacific, the extremely low effective population size estimated during the bottleneck (*N*_b_ = 111) is lower than the minimal estimate of 500 thought to be required for a population to be able to adapt to environmental changes or pressures (Frankham, Briscoe, & Ballou, 2010). While this tiger shark population seems to have expanded since the bottleneck, we were not able to estimate the current effective population size, and this population may well be vulnerable in terms of genetic diversity. For now, the tiger shark is classified as Near Threatened in the IUCN Red List of threatened species (Simpfendorfer, 2009). Its conservation status might need to be revised according to the results highlighted in this and previous studies.

## 5 CONCLUSION

The study presented here confirms the genetic connectivity of tiger sharks within the western Indo‐Pacific, using both microsatellites and mitochondrial markers. While mitochondrial differentiation estimates were slightly higher than those of microsatellite, further analyses are needed to confirm whether differentiation reflects female philopatry to specific nurseries. Individuals from the Indo‐Pacific form a discrete population. Management and conservation programmes thus need to be designed at such scales in order to maximize potential efficacy. Meanwhile, localized intensive fishing may yet stand to impact the whole population. Furthermore, the detection of low genetic diversity as well as a recent bottleneck (in the Holocene), during which the effective population size of tiger sharks in this region may have dropped as low as 111 individuals, points to a potentially resultant vulnerable population. Further assessments of the health status of this population as well as conservation plans are thus particularly needed to conserve the tiger shark within the western Indo‐Pacific. Its conservation status might need to be revised toward a higher vulnerability level, as the ability of tiger sharks to withstand high levels of fishing pressure might be lower than previously thought.

## CONFLICT OF INTEREST

None declared.

## AUTHOR CONTRIBUTIONS

GC, EC, BJH, NEH, JEGN, AJT, PB, LV, and SJ provided samples. AP and HM did lab work. AP, HM, and VR analyzed the data. All authors contributed to the writing of the manuscript.HM and SJ designed research.

## Data Availability

Microsatellite genotypes: Dryad https://doi.org/10.5061/dryad.3161qp0 Mitochondrial sequences: GenBank Accession Numbers: MK359166‐MK359171 and MK582583‐MK582596.
