# Supplementary material for: Genetic population structure and demography of an apex predator, the tiger shark Galeocerdo cuvier
Source: Ecol Evol. 2019 May 4;9(10):5551–71. doi: 10.1002/ece3.5111 (PMC6540675; doi:10.1002/ece3.5111)
Supplement: Supplementary file 1 [file ECE3-9-5551-s001.docx]

**Supplemental Information for:**

**Genetic population structure and demography of an apex predator, the tiger shark *Galeocerdo cuvier***

Agathe Pirog, Sébastien Jaquemet, Virginie Ravigné, Geremy Cliff, Eric Clua, Bonnie J. Holmes, Nigel E. Hussey, John E. G. Nevill, Andrew J. Temple, Per Berggren, Laurent Vigliola, Hélène Magalon

**Table of contents**

**Appendix S1.** Panels used to multiplex loci post-PCR and allelic ranges. 2

**Appendix S2.** Allelic frequencies at the eight microsatellite studied in common in the present study (red square) and the study of Holmes *et al* (2017). 3

**Appendix S3.** Inference of demographic history using Approximate Bayesian Computation (ABC) and performed with Diyabc v.2.1.0 4

**Appendix S4.** Summary statistics for each sampling locality for the three mitochondrial markers used, the control region (*CR*, 862 bp), *COI* (652 pb) and *cytb* (931 bp). 7

**Appendix S5.** Posterior trace file of the BEAST analysis. 8

**Appendix S6.** Tiger shark mitochondrial *CR-COI-cytb* haplotypes identified in each locality sampled. 9

**Appendix S7.** Bayesian information criterion (BIC) values versus the number of clusters assumed for the DAPC analyses performed for the 27‑msat dataset, the 8‑msat dataset, Holmes 8‑msat dataset and Bernard 8‑msat dataset. 10

**Appendix S8.** DAPC analyses performed for the 8‑msat dataset, for (a) three clusters assumed, (b) four clusters assumed, (c) five clusters assumed. 11

**Appendix S9.** *Galeocerdo cuvier* demography statistics (Tajima’s *D* and Fu’s *F_S_*) for the concatenated mitochondrial sequence *CR-COI-cytb* 12

**Appendix S10.** Fit of observed data to models selected using ABC analysis and scenario comparison using samples from Reunion Island. 13

**Appendix S11.** Posterior distributions of the demographic parameters drawn with Scenario 6 using samples from Reunion Island. 14

**Appendix S12.** Fit of observed data to models selected using ABC analysis and scenario comparison using samples from New Caledonia. 15

**Appendix S13.** Posterior distributions of the demographic parameters drawn with Scenario 6 using samples from New Caledonia. 16

**Appendix S14.** Characteristics of demographic parameter posterior distributions estimated using *Galeocerdo cuvier* samples from New Caledonia with Diyabc under Scenario 6. 17

**Appendix S1**. Panels used to multiplex loci post-PCR and allelic ranges (in base pairs, without M13-tail).

| Locus Name | Panel | Fluorochrome | Allelic range | Reference |
| --- | --- | --- | --- | --- |
| Gc02 | 1 | VIC | 93-105 | Pirog et al. 2016 |
| Gc03 | 1 | PET | 119-129 | Pirog et al. 2016 |
| TGR1185 | 1 | 6-FAM | 145-161 | Bernard et al.2015 |
| Cl14 | 1 | VIC | 184-218 | Pirog et al. 2015 |
| TGR212 | 1 | NED | 194-200 | Bernard et al.2015 |
| Cl12 | 2 | 6-FAM | 105-125 | Pirog et al. 2015 |
| TGR348 | 2 | VIC | 107-193 | Bernard et al.2015 |
| Gc04 | 2 | 6-FAM | 195-199 | Pirog et al. 2016 |
| Cli100 | 2 | NED | 207-227 | Keeney et al. 2003 |
| Gc05 | 3 | PET | 105-123 | Pirog et al. 2016 |
| Gc01 | 3 | VIC | 135-147 | Pirog et al. 2016 |
| Cl17 | 3 | 6-FAM | 167-181 | Pirog et al. 2015 |
| TGR943 | 3 | NED | 139-215 | Bernard et al.2015 |
| TIG15 | 3 | PET | 209-256 | Mendes et al. 2016 |
| TIG17 | 3 | VIC | 251-269 | Mendes et al. 2016 |
| Gc07 | 4 | VIC | 114-146 | Pirog et al. 2016 |
| TGR1157 | 4 | PET | 196-226 | Bernard et al.2015 |
| TGR891 | 4 | NED | 336-408 | Bernard et al.2015 |
| Gc06 | 5 | 6-FAM | 116-122 | Pirog et al. 2016 |
| TGR47 | 5 | PET | 150-162 | Bernard et al.2015 |
| TGR1033 | 5 | NED | 164-180 | Bernard et al.2015 |
| Gc08 | 5 | VIC | 207-225 | Pirog et al. 2016 |
| TIG01 | 6 | 6-FAM | 127-135 | Mendes et al. 2016 |
| TIG07 | 6 | VIC | 151-169 | Mendes et al. 2016 |
| TIG10 | 6 | NED | 248-258 | Mendes et al. 2016 |
| TIG19 | 6 | 6-FAM | 321-335 | Mendes et al. 2016 |
| TIG12 | 6 | VIC | 342-366 | Mendes et al. 2016 |

# Appendix S2. Allelic frequencies at the eight microsatellite studied in common in the present study (red square) and the study of Holmes *et al* (2017). (ZAN, Zanzibar; SEY, Seychelles; SAF, South Africa; MAD, Madagascar; RUN, Reunion Island; AS, Andaman Sea; AUS1, Western Australian coast; AUS2, Queensland, Australia; AUS3, New South Wales, Australia; AUS4, Northern Territories, Australia; COR, Coral Sea; NCA, New Caledonia; HAW, Hawaii; GOM, Gulf of Mexico; FLE, Florida East Coast; BAH, Bahamas; USVI, US Virgin Islands; BRA, Brazil)


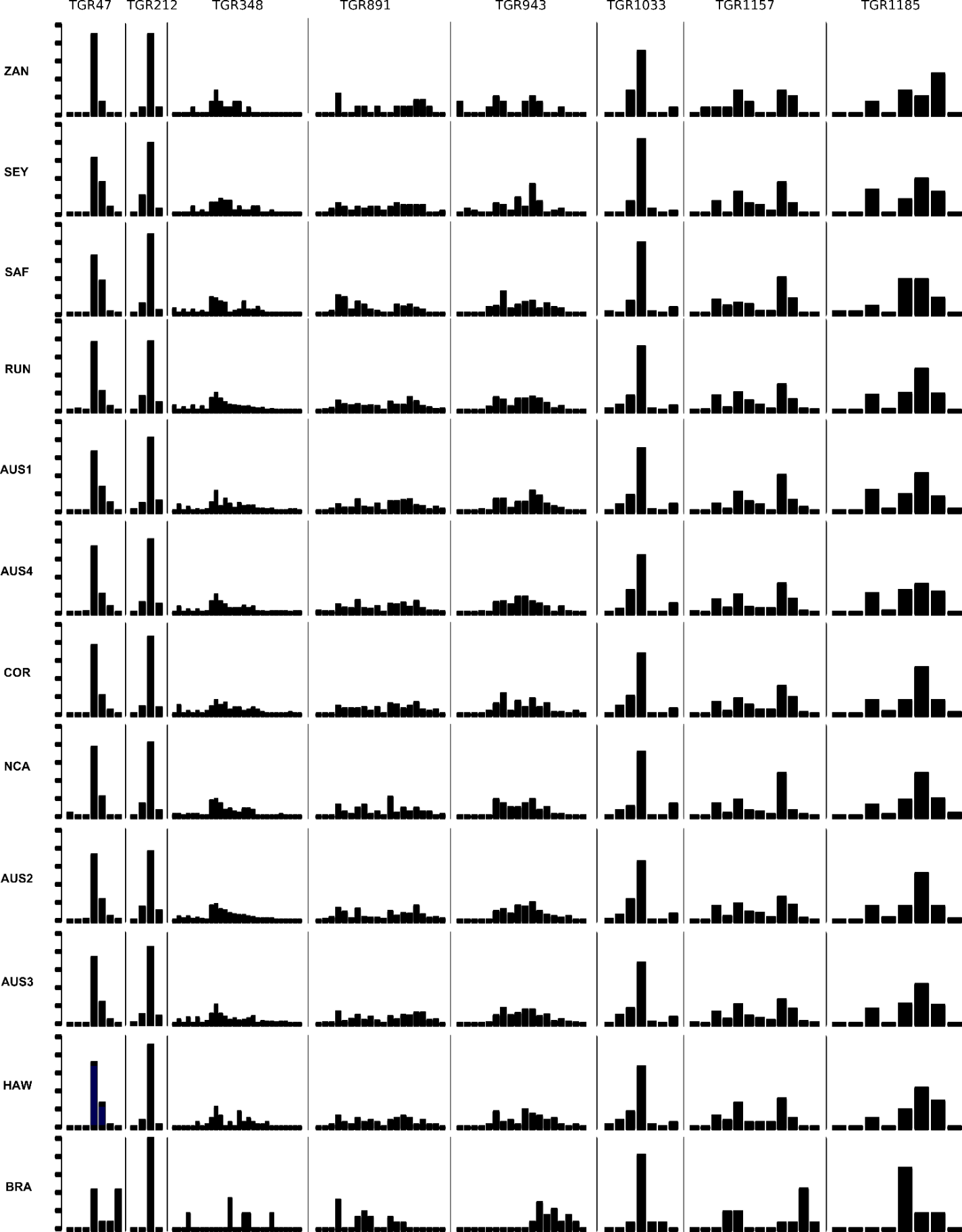


# Appendix S3. Inference of demographic history using Approximate Bayesian Computation (ABC) and performed with Diyabc v.2.1.0

To identify effective population size variations in the Indo-Pacific, we used a coalescent framework through approximate Bayesian computation (ABC) using Diyabc v.2.1.0 (Cornuet et al., 2014) with both the 27‑msat dataset and the *CR‑COI‑cytb* dataset.

*Scenarios*

We defined *N_0_* the actual effective population size, *N_1_* the ancestral effective population size, *N_b_* the effective population size during a bottleneck, and *N_e_* the effective population size during an expansion. Then seven scenarios were set up to test for: (Scenario 1) a recent (less than 500 generations) decrease (*N_0_* < *N_1_*), (Scenario 2) a more ancient (between 10^3^ and 5×10^5^ generations in the past) decrease, (Scenario 3) a recent (less than 500 generations) expansion (*N_0_* > *N_1_*), (Scenario 4) a more ancient (between 10^3^ and 5×10^5^ generations in the past) expansion, (Scenario 5) an expansion followed by a decrease (*N_e_ > N_0_,N_1_*), (Scenario 6) a bottleneck (*N_b_ <N_0_,N_1_*) and (Scenario 7) a constant effective population size (*N_0_* = *N_1_*) (Figure 2). For Scenarios 5 and 6, the end of the expansion/bottleneck was set at 5 generations in the past, which approximately corresponds to the interdiction of commercial exploitation of the tiger shark in Reunion Island (in 1999). Generation time was supposed to be around 7 -10 years (Branstetter, Musick, & Colvocoresses, 1987; Holmes et al., 2015; Kneebone, Natanson, Andrews, & Howell, 2008; Wintner & Dudley, 2000).

*Prior distribution*

Parameters were drawn in the prior distributions described in the enclosed Table S1. Because of the lack of knowledge on effective sizes of populations of *G. cuvier*, ranges were chosen broad. For population sizes and times of effective population size variations, uniform distributions with large interval were chosen. Parameters for microsatellite mutation models were set by default following a generalized stepwise mutation model.

**Table S1.** Prior distributions of parameters used in the Diyabc analysis.

| Parameters | Correspondence in scenarios | Priors |
| --- | --- | --- |
| *N_high_* | *N_1_* for Scenarios 3 and 4 | uniform [10^2^, 10^4^] |
| *N_low_* | *N_1_* for Scenarios 1 and 2 | uniform [10, 5×10^2^] |
| *N_now_* | *N_0_* for all scenarios | uniform [10, 10^4^] |
| *N_e_* | *N_e_* for Scenario 5 | uniform [10, 10^4^] |
| *N_b_* | *N_b_* for Scenario 6 | uniform [10, 5×10^3^] |
| *N_1_* | *N_1_* for Scenarios 5 and 6 | uniform [10, 10^4^] |
| *t_1_* (in generations) | beginning of decrease or expansion for Scenarios 1 and 3 | uniform [5, 5×10^2^] |
| *t_2_* (in generations) | beginning of decrease or expansion for Scenarios 2 and 4 | uniform [10^3^, 5×10^5^] |
| *t* (in generations) | *t* | uniform [1, 10^3^] |
| *µ_seq_* | Mean of the gamma distribution of locus specific mutation rates | uniform [10^-9^, 10^-4^] |
| *µ_sat_* | Mean of the gamma distribution of locus specific mutation rates | uniform [10^-6^, 10^-2^] |
| Constraint on parameters | *N_high_>N_now_*; *N_low_<N_now_*; *N_e_>N_now_*; *N_e_>N_1_*; *N_b_<N_1_*; *N_b_<N_now_* |  |

*Summary statistics*

The genetic variation within the population was summarized using a set of 12 summary statistics, all the ones available in Diyabc v.2.1.0 (Cornuet et al., 2014). Choosing all summary statistics is not recommended, but only few are available when studying only one population, and we thus privileged the estimate quality. We thus used, for the 27 microsatellite loci, the mean number of alleles over loci *K*, the mean of Nei’s genetic diversity *H*, the mean size variance and the mean Garza-Williamson index *M.* For the concatenated mitochondrial sequence, we used the number of haplotypes *H*, the number of segregating sites *S*, the mean number of pairwise differences *Pi*, the variance of the number of pairwise differences, Tajima’s *D*, the mean of numbers of the rarest nucleotide at segregating sites and the variance of numbers of the rarest nucleotide at segregating sites.

*Prior validation*

Prior choices were validated performing a principal component analysis (PCA) in the space of summary statistics for the first 100,000 datasets simulated with parameter values drawn from the prior parameters. The observed dataset was then projected as a supplementary individual to determine (visual check) whether it fell well within the variability of simulated data (Cornuet, Ravigné, & Estoup, 2010) (Figure S2).

*Implementing ABC procedure*

For each scenario, we performed 10^6^ simulations each using a different set of parameters sampled from the prior distributions. For each simulated dataset, the summary statistics were computed. This allowed building a reference table with 7×10^6^ lines containing the number of the simulated scenario, the set of sampled parameter values and the corresponding summary statistics. From this reference table, posterior probabilities were computed via logistic regression on the 1% of simulated data sets closest to the empirical data (Cornuet et al., 2008). Summary statistics were transformed by linear discrimination analysis prior to logistic regression to reduce correlation among explanatory variables and provide conservative estimates of scenario discrimination (Estoup et al., 2012). Posterior distributions of all parameters were then estimated from the selected model, based on the 1% of simulated data sets closest to the empirical data.

Then the confidence in scenario choice was performed by simulating pseudo-observed datasets drawing (with replacement) the scenario ID and parameter values from the 500 simulated datasets closest to the observed dataset (i.e. the 500 datasets of the reference table with the smallest Euclidian distance). For each of the pseudo observed dataset produced this way, we applied the same procedure than the one performed to select the best scenario, to estimate their respective posterior probabilities and measure the proportion of times the right scenario has the highest posterior probability. The posterior error rate is then given as a proportion of wrongly identified scenarios over the test datasets for both the direct ad logistic approaches.

Finally, bias and mean square error of parameter estimations were computed by simulating 500 pseudo-observed datasets under the retained scenario (Scenario 6), and by comparing the real and estimated values of parameters. The parameters values of the pseudo-observed datasets were drawn from the posterior distributions estimated using a standard ABC procedure.

*References*

Branstetter, S., Musick, J. A., & Colvocoresses, J. A. (1987). A comparison of the age and growth of the tiger shark, *Galeocerdo cuvieri*, from off Virginia and from the Northwestern Gulf of Mexico. *Fishery Bulletin*, *85*(2), 269-279.

Cornuet, J.-M., Pudlo, P., Veyssier, J., Dehne-Garcia, A., Gautier, M., Leblois, R., . . . Estoup, A. (2014). DIYABC v2.0: A software to make approximate Bayesian computation inferences about population history using single nucleotide polymorphism, DNA sequence and microsatellite data. *Bioinformatics*, *30*(8), 1187-1189. doi: 10.1093/bioinformatics/btt763

Cornuet, J.-M., Ravigné, V., & Estoup, A. (2010). Inference on population history and model checking using DNA sequence and microsatellite data with the software DIYABC (v1.0). *BMC Bioinformatics*, *11*(1), 401. doi: 10.1186/1471-2105-11-401

Cornuet, J.-M., Santos, F., Beaumont, M. A., Robert, C. P., Marin, J.-M., Balding, D. J., . . . Estoup, A. (2008). Inferring population history with DIYABC: A user-friendly approach to approximate Bayesian computation. *Bioinformatics*, *24*(23), 2713-2719. doi: 10.1093/bioinformatics/btn514

Estoup, A., Lombaert, E., Marin, J.-M., Guillemaud, T., Pudlo, P., Robert, C. P., & Cornuet, J.-M. (2012). Estimation of demo-genetic model probabilities with Approximate Bayesian Computation using linear discriminant analysis on summary statistics. *Molecular Ecology Resources*, *12*(5), 846-855. doi: 10.1111/j.1755-0998.2012.03153.x

Holmes, B. J., Peddemors, V. M., Gutteridge, A. N., Geraghty, P. T., Chan, R. W. K., Tibbetts, I. R., & Bennett, M. B. (2015). Age and growth of the tiger shark *Galeocerdo cuvier* off the East coast of Australia. *Journal of Fish Biology*, *87*(2), 422-448. doi: 10.1111/jfb.12732

Kneebone, J., Natanson, L. J., Andrews, A. H., & Howell, W. H. (2008). Using bomb radiocarbon analyses to validate age and growth estimates for the tiger shark, *Galeocerdo cuvier*, in the Western North Atlantic. *Marine Biology*, *154*(3), 423-434. doi: 10.1007/S00227-008-0934-Y

Wintner, S. P., & Dudley, S. F. J. (2000). Age and growth estimates for the tiger shark, *Galeocerdo cuvier*, from the East coast of South Africa. *Marine and Freshwater Research*, *51*(1), 43-53. doi: 10.1071/MF99077

# Appendix S4. Summary statistics for each sampling locality (ZAN, Zanzibar; SEY, Seychelles; SAF, South Africa; MAD, Madagascar; RUN, Reunion Island; AUS1, Western Australian coast; AUS2, Queensland, Australia; NCA, New Caledonia) for the three mitochondrial markers used, the control region (*CR*, 862 bp), *COI* (652 pb) and *cytb* (931 bp): *N_s_*, number of individuals sequenced; *H*, number of haplotypes, *h*, haplotype diversity; *S*, number of polymorphic sites; *π*, nucleotide diversity. In parentheses, are indicated standard errors.

| *CR* | ZAN | SEY | SAF | RUN | AUS1 | AUS2 | NCA | TOT |
| --- | --- | --- | --- | --- | --- | --- | --- | --- |
| *N_S_* | 8 | 20 | 24 | 103 | 9 | 10 | 25 | 199 |
| *H* | 2 | 4 | 4 | 6 | 4 | 3 | 3 | 9 |
| *S* | 1 | 3 | 3 | 5 | 3 | 2 | 2 | 7 |
| *h* | 0.43 (0.06) | 0.57 (0.02) | 0.37 (0.02) | 0.34 (0.01) | 0.78 (0.04) | 0.69 (0.03) | 0.56 (0.01) | 0.48 (0.00) |
| *π* | 0.00049 (0.00020) | 0.00081 (0.00016) | 0.00046 (0.00010) | 0.00047 (0.00005) | 0.00115 (0.00032) | 0.00095 (0.00026) | 0.00069 (0.00013) | 0.00068 (0.00000) |
| *COI* | ZAN | SEY | SAF | RUN | AUS1 | AUS2 | NCA | TOT |
| *N_S_* | 8 | 24 | 30 | 43 | 9 | 10 | 23 | 147 |
| *H* | 1 | 1 | 2 | 2 | 1 | 1 | 2 | 4 |
| *S* | 0 | 0 | 1 | 1 | 0 | 0 | 1 | 3 |
| *h* | 0.00 (0.00) | 0.00 (0.00) | 0.07 (0.01) | 0.21 (0.01) | 0.00 (0.00) | 0.00 (0.00) | 0.09 (0.02) | 0.09 (0.00) |
| *π* | 0.00000 (0.00000) | 0.00000 (0.00000) | 0.00010 (0.00004) | 0.00032 (0.00007) | 0.00000 (0.00000) | 0.00000 (0.00000) | 0.00013 (0.00006) | 0.00014 (0.00000) |
| *cytb* | ZAN | SEY | SAF | RUN | AUS1 | AUS2 | NCA | TOT |
| *N_S_* | 8 | 22 | 23 | 39 | 9 | 10 | 19 | 130 |
| *H* | 1 | 6 | 3 | 5 | 2 | 2 | 7 | 14 |
| *S* | 0 | 5 | 2 | 4 | 1 | 1 | 7 | 12 |
| *h* | 0.00 (0.00) | 0.41 (0.03) | 0.42 (0.02) | 0.57 (0.01) | 0.22 (0.06) | 0.20 (0.05) | 0.61 (0.03) | 0.47 (0.00) |
| *π* | 0.00000 (0.00000) | 0.00066 (0.00013) | 0.00048 (0.00010) | 0.00070 (0.00010) | 0.00024 (0.00012) | 0.00022 (0.00010) | 0.00099 (0.00018) | 0.00063 (0.00001) |

# Appendix S5. Posterior trace file of the BEAST analysis. Concatenated runs of the five analyses performed are shown, as well as the Effective Sample Sizes (ESS) of the posterior distribution.


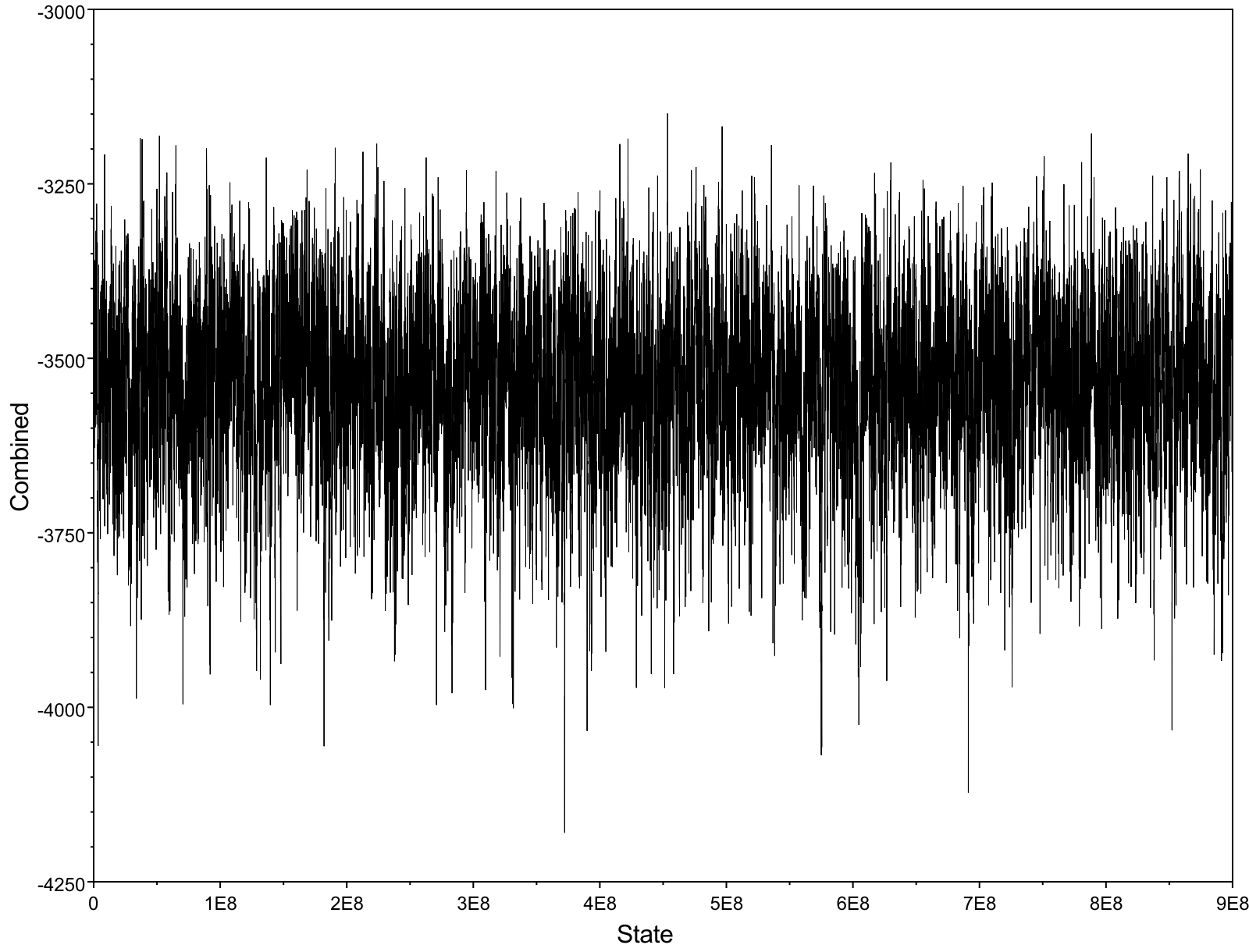


**ESS = 1770**

# Appendix S6. Tiger shark mitochondrial *CR-COI-cytb* haplotypes identified in each locality sampled. Sizes of each circle represent the number of mitochondrial sequences obtained. The three main haplotypes identified are represented in black, dark grey and light grey. In white are all the other haplotypes.

**
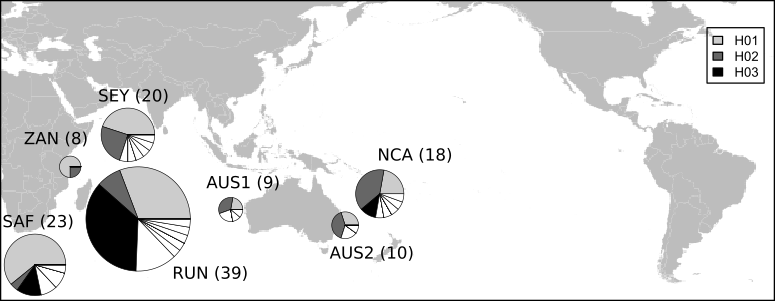
**

# Appendix S7. Bayesian Information Criterion (BIC) values versus the number of clusters assumed for the DAPC analyses performed for the 27‑msat dataset, the 8‑msat dataset, Holmes 8‑msat dataset and Bernard 8‑msat dataset.


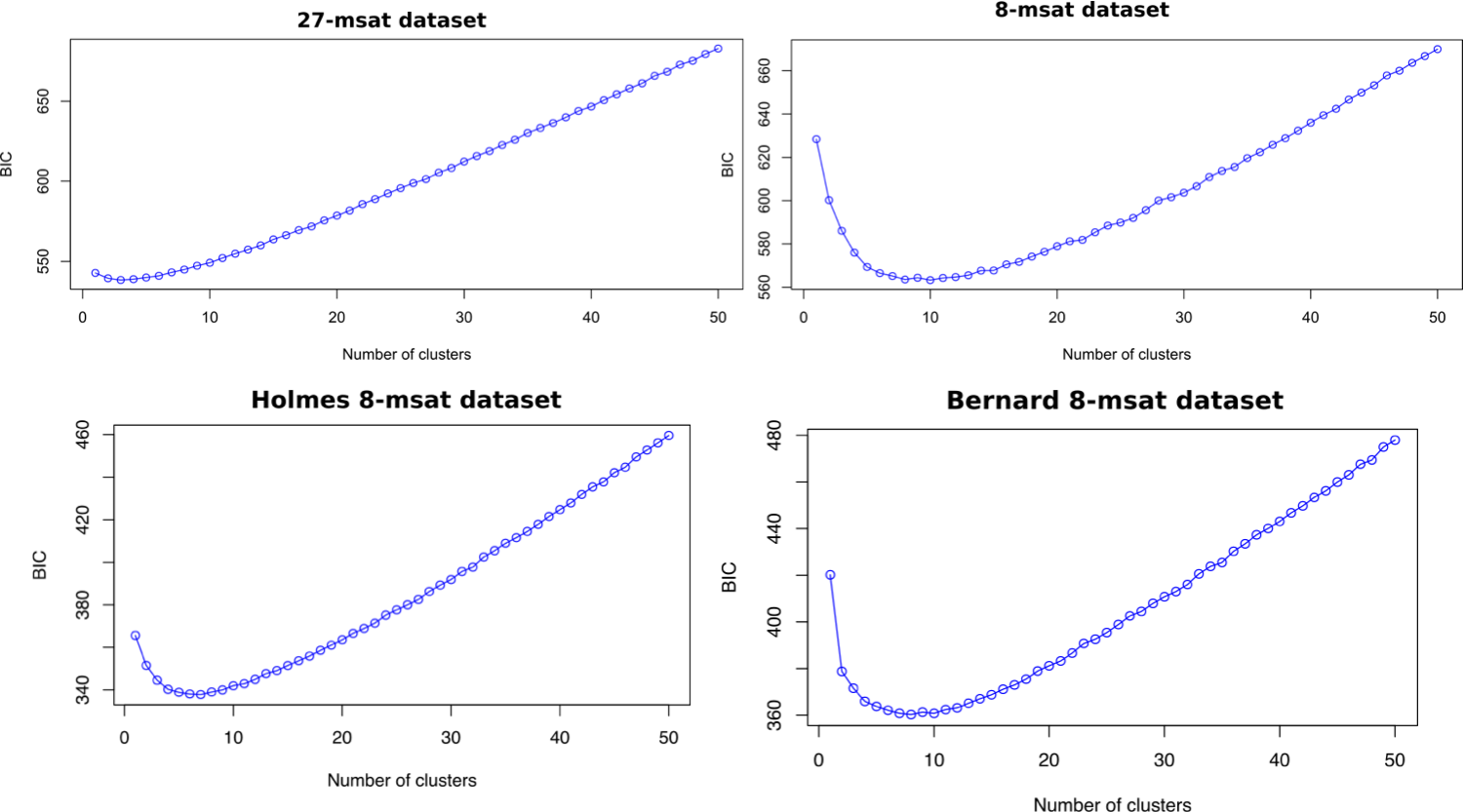


# Appendix S8. DAPC analyses performed for the 8‑msat dataset, for (a) three clusters assumed, (b) four clusters assumed, (c) five clusters assumed.


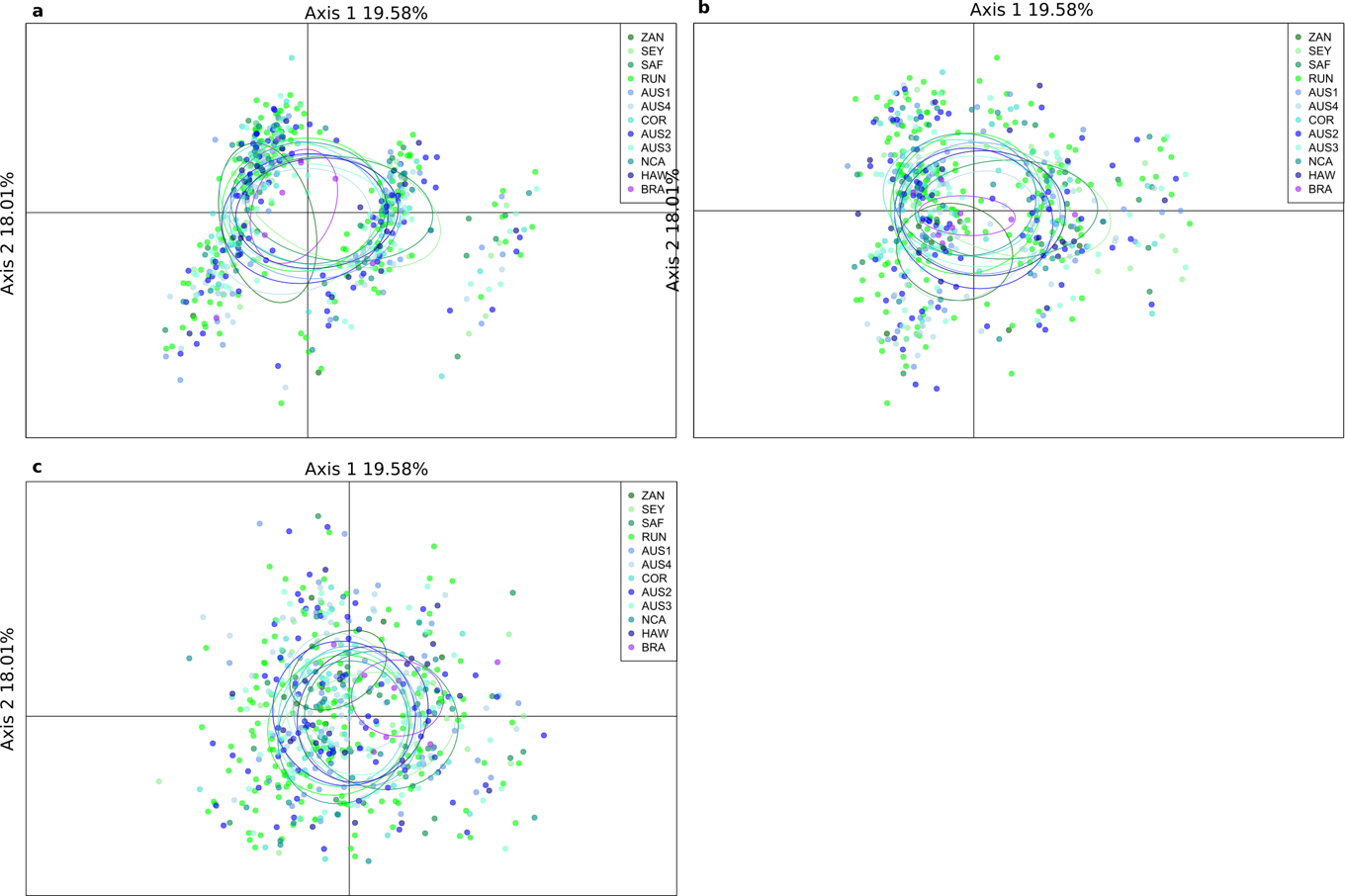


# Appendix S9. *Galeocerdo cuvier* demography statistics (Tajima’s *D* and Fu’s *F_S_*) for the concatenated mitochondrial sequence *CR-COI-cytb* (Zanzibar, ZAN; Seychelles, SEY; South Africa, SAF; Reunion Island, RUN; Western Australian coast, AUS1; North-Eastern Australian coast, AUS2; New Caledonia, NCA); *: *P* < 0.05, **: *P* < 0.01, ***: *P*< 0.001

|  | *D* | *F_S_* |
| --- | --- | --- |
| *Western Indian Ocean* | −**1.52** | −**11.34***** |
| ZAN | 0.33 | 0.54 |
| SEY | −1.44 | −3.88** |
| SAF | −1.07 | −2.50* |
| RUN | −1.03 | −3.74* |
| *Australia and Western Pacific* | −**1.56** | −**4.74**** |
| AUS1 | −0.69 | −1.99* |
| AUS2 | −0.13 | −0.83 |
| NCA | −1.50 | −3.56** |
| *All localities pooled* | −**1.83**** | −**18.12***** |

# Appendix S10. Fit of observed data to models selected using ABC analysis and scenario comparison using samples from Reunion Island. (a) Plane of the principle components analysis was generated from 70 summary statistics from simulations, using prior distributions of historical parameters: open circles are simulated data sets, generated using prior distributions of historical parameters; closed yellow circle is the observed data set. Results of model testing with (b) direct estimate of posterior probability (c) logistic regression estimate of posterior probability; ordinate is probability, abscissa is number of simulated data sets.

**
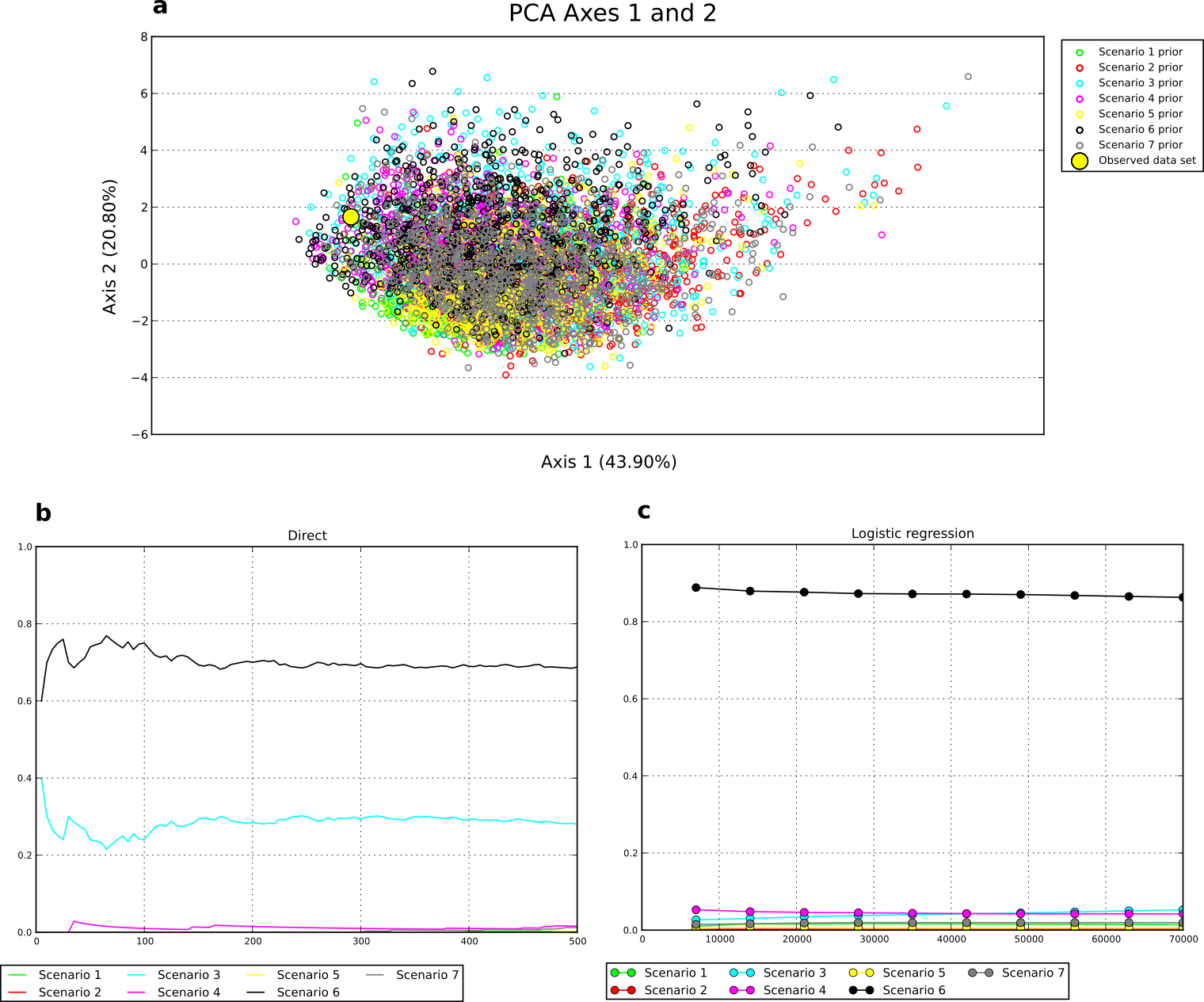
**

# Appendix **S11.** Posterior distributions of the demographic parameters drawn with Scenario 6 using samples from Reunion Island. *N_0_*, the actual effective population size; *N_1_*, the ancestral effective population size; *N_b_*, the effective population size during a bottleneck; *t*, beginning of the bottleneck period.

**
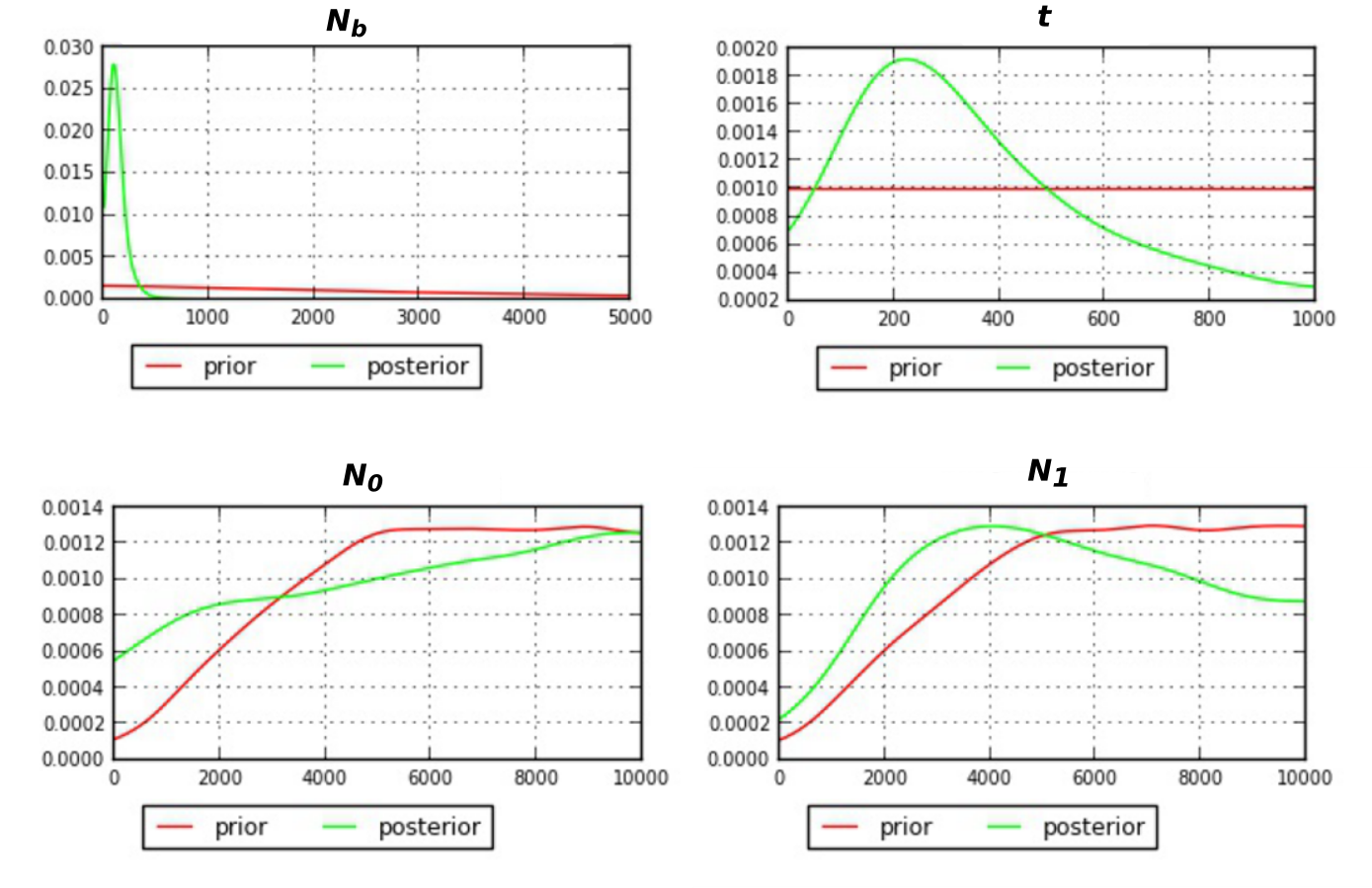
**

# Appendix S12. Fit of observed data to models selected using ABC analysis and scenario comparison using samples from New Caledonia. (a) Plane of the principle components analysis was generated from 70 summary statistics from simulations, using prior distributions of historical parameters: open circles are simulated data sets, generated using prior distributions of historical parameters; closed yellow circle is the observed data set. Results of model testing with (b) direct estimate of posterior probability (c) logistic regression estimate of posterior probability; ordinate is probability, abscissa is number of simulated data sets.

**
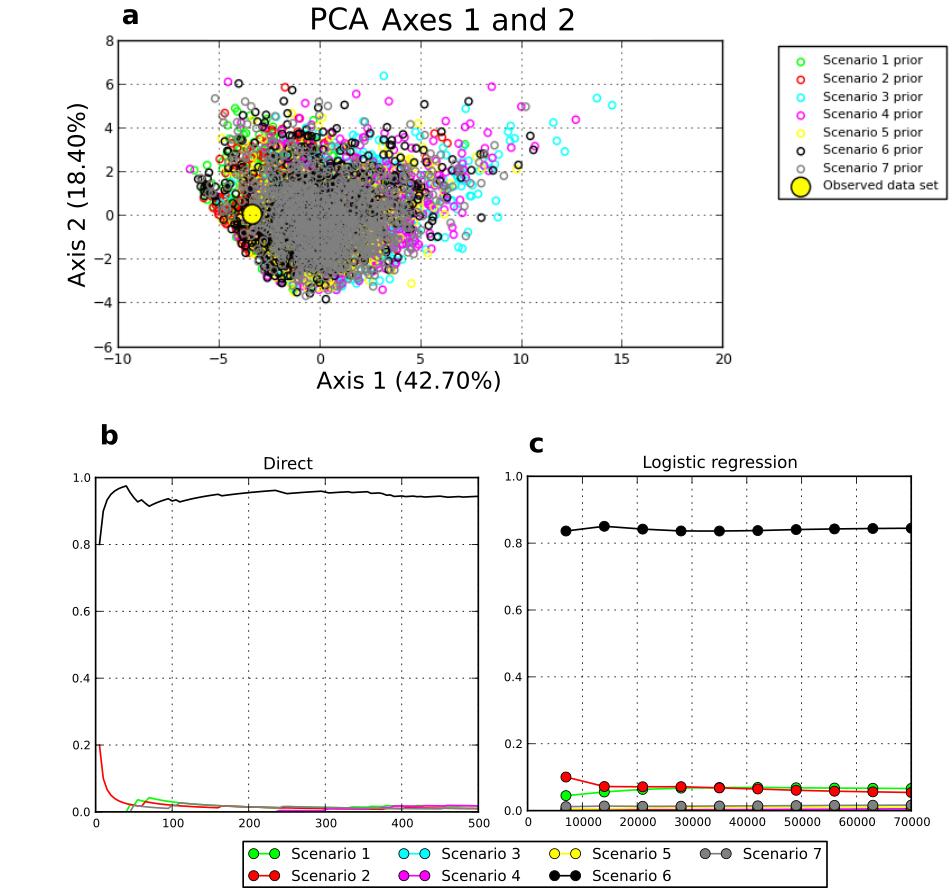
**

# Appendix S13. Posterior distributions of the demographic parameters drawn with Scenario 6 using samples from New Caledonia. *N_0_*, the actual effective population size; *N_1_*, the ancestral effective population size; *N_b_*, the effective population size during a bottleneck; *t*, beginning of the bottleneck period.

**
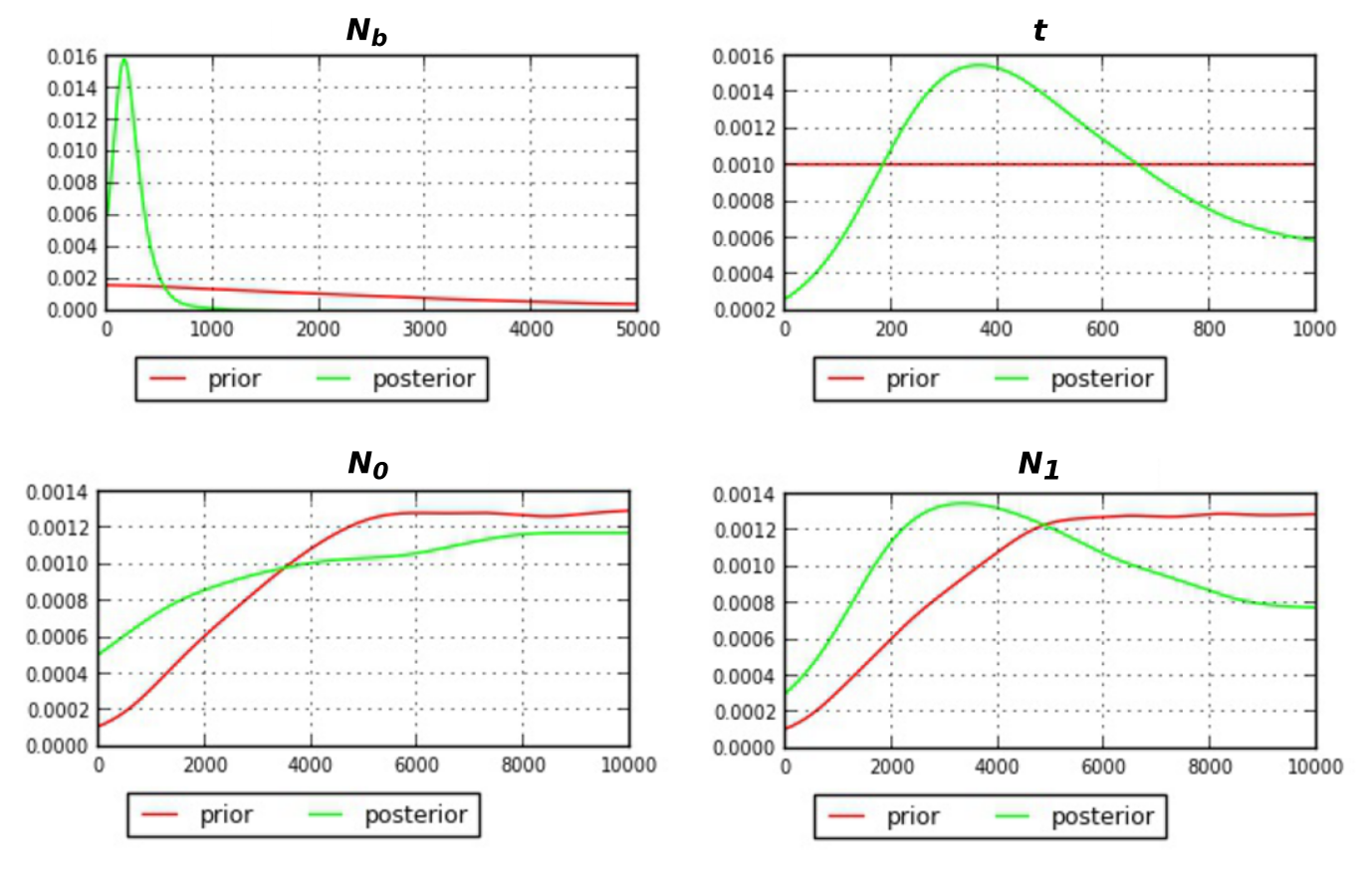
**

# Appendix S14. Characteristics of demographic parameter posterior distributions estimated using *Galeocerdo cuvier* samples from New Caledonia with Diyabc under Scenario 6. *N_0_*, the actual effective population size; *N_1_*, the ancestral effective population size; *N_b_*, the effective population size during a bottleneck; *t*, beginning of the bottleneck period; RMSE, Root Mean Square Error.

| Parameter | median | 2.5% quantile | 97.5% quantile | RMSE |
| --- | --- | --- | --- | --- |
| *N_b_* | 193 | 69 | 776 | 0.917 |
| *t* | 458 | 99 | 954 | 1.127 |
| *N_0_* | 5660 | 694 | 9800 | 2.067 |
| *N_1_* | 4750 | 989 | 9670 | 1.32 |
